# Astroglial modulation of synaptic function in the non-demyelinated cerebellar cortex is dependent on MyD88 signaling in a model of toxic demyelination

**DOI:** 10.1186/s12974-025-03368-9

**Published:** 2025-02-23

**Authors:** Melanie Lohrberg, Lena Sünke Mortensen, Carolina Thomas, Franziska Fries, Franziska van der Meer, Alexander Götz, Carolin Landt, Hong Jun Rhee, JeongSeop Rhee, David Gómez-Varela, Manuela Schmidt, Wiebke Möbius, Torben Ruhwedel, Luis A. Pardo, Linus Remling, Nadine Kramann, Claudia Wrzos, Erik Bahn, Christine Stadelmann, Alonso Barrantes-Freer

**Affiliations:** 1 Department of Neuropathology, University Medical Center Göttingen, Göttingen, Germany; 2 Campus Institute Data Science, Göttingen, Germany; 3 Interdisciplinary Center for Bioinformatics (IZBI), University of Leipzig, Leipzig, Germany; 4 Paul-Flechsig-Institute of Neuropathology, University Medical Center Leipzig, Leipzig, Germany; 5 Department of Molecular Neurobiology, Max-Planck Institute for Multidisciplinary Sciences, Göttingen, Germany; 6 Division of Pharmacology and Toxicology, Department of Pharmaceutical Sciences, University of Vienna, Vienna, Austria; 7 Department of Neurogenetics, Max-Planck Institute for Multidisciplinary Sciences, Göttingen, Germany; 8 Oncophysiology Group, Max-Planck Institute for Multidisciplinary Sciences, Göttingen, Germany; 9 Cluster of Excellence “Multiscale Bioimaging: From Molecular Machines to Network of Excitable Cells” (MBExC), University of Göttingen, Göttingen, Germany

## Abstract

Progressive neurological decline in multiple sclerosis is associated with axonal loss and synaptic dysfunction in the non-demyelinated normal appearing gray matter (NAGM) and prominently in the cerebellum. In contrast to early disease stages, where synaptic and neuro-axonal pathology correlates with the extent of T cell infiltration, a prominent role of the innate immune system has been proposed for progressive MS. However, the specific contribution of microglia and astrocytes to synaptic cerebellar pathology in the NAGM– independent of an adaptive T cell response - remains largely unexplored. In the present study, we quantified synaptic changes in the cerebellar NAGM distant from demyelinated lesions in a mouse model of toxic demyelination. Proteomic analysis of the cerebellar cortex revealed differential regulation of synaptic and glutamate transport proteins in the absence of evident structural synaptic pathology or local gray matter demyelination. At the functional level, synaptic changes manifested as a reduction in frequency-dependent facilitation at the parallel fiber– Purkinje cell synapse. Further, deficiency of MyD88, an adaptor protein of the innate immune response, associated with a functional recovery in facilitation, reduced changes in the differential expression of synaptic and glutamate transport proteins, and reduced transcription levels of inflammatory cytokines. Nevertheless, the characteristics of demyelinating lesions and their associated cellular response were similar to wild type animals. Our work brings forward an experimental paradigm mimicking the diffuse synaptic pathology independent of demyelination in late stage MS and highlights the complex regulation of synaptic pathology in the cerebellar NAGM. Moreover, our findings suggest a role of astrocytes, in particular Bergmann glia, as key cellular determinants of cerebellar synaptic dysfunction.

**Supplementary Information:**

The online version contains supplementary material available at 10.1186/s12974-025-03368-9.

## Introduction

Multiple sclerosis (MS) is characterized by inflammatory demyelinated lesions of the white and grey matter, as well as diffuse progressive neurodegeneration [1–9]. The involvement of the cerebellum in MS pathology is a prominent feature figuring in the earliest descriptions of the disease, and is associated with a broad spectrum of symptoms ranging from altered motor skills to cognitive impairment [6, 10, 11].

It is generally accepted that during relapses, clinical symptoms are mainly attributed to the infiltration of inflammatory cells, causing demyelinated lesions within the white matter. However, extensive neurodegeneration is observed in the grey matter (GM) (reviewed in [12]). GM alterations, such as neuronal atrophy and loss, synaptic loss and neurite loss are apparent in demyelinated lesions. Interestingly, however, these alterations are to a similar degree also found in the non-demyelinated normal-appearing GM (NAGM) [1, 2, 4, 7, 13, 14]. Synaptic loss in the NAGM of MS patients is more pronounced than neuronal loss, indicating primary synaptic pathology [1–5, 7]. Moreover, a reduction in dendritic density in the NAGM has been demonstrated using high-resolution microscopy in patients with long-standing MS. This dendrite loss was independent of nearby cortical demyelination and axonal loss and seemed to be a consequence of diffuse activation of the complement cascade and subsequent microglial activation [5]. Even though different studies tackle GM pathology in MS and related animal models, cerebellar NAGM pathology remains largely unexplored.

The cerebellum harbors different neuronal populations, whose neurites form a complex circuit assuring motor coordination. The most abundant neuron is the cerebellar granule cell that gives rise to T-shaped axons forming the so-called parallel fibers that build synapses on the dendritic tree of Purkinje cells (PCs) [15, 16]. The parallel fiber-Purkinje cell (PF-PC) synapses use glutamate as a neurotransmitter and are involved in the pathology of different disorders (reviewed in [15]). Although astrocytic coverage of synapses is a general phenomenon in the CNS and essential for metabolic support and functionality of synapses, coverage is even more pronounced in case of PF-PC synapses, as 65–87% of excitatory synapses on PCs are ensheathed by astrocytic processes. The ensheathment is formed by Bergmann glia (BG), a specialized astrocytic population that has its soma within or in close proximity to the PC-layer and branches into the molecular layer of the cerebellar cortex [17, 18]. Glutamate transporters are abundantly expressed in the astrocytic plasma membrane and are essential for synaptic function, as they clear released glutamate from the synaptic cleft [17, 19]. Studies indicate that the L-glutamate/L-aspartate transporter (GLAST/EAAT1, human/murine nomenclature) is ubiquitously expressed throughout the cerebellum, whereas other transporters are expressed in a cell specific manner. Besides GLAST, the glutamate transporter-1 (GLT-1/EAAT2) that is primarily found on BG, as well as the excitatory amino acid transporter 4 (EAAT4) that is mostly detectable on PCs, are involved in glutamate homeostasis within the cerebellum [17, 20–27].

PCs are crucial for the coordinative function of the cerebellum, as they unite different signals within the cerebellar cortex and give rise to axons that are the sole output route from the cerebellar cortex to the cerebellar nuclei. Consequently, PC loss or dysfunction is associated with cerebellar ataxia, which can occur as a consequence of MS or other neurodegenerative or genetic disorders (reviewed in [15]). Recent studies indicated that progressive degenerative processes in BG (including reactive morphology, decreased GLT-1 expression and activation of the metabolic stress response) lead to an impaired glutamate uptake and insufficient metabolic support of PCs, finally provoking PC excitotoxicity in a mouse model of spinocerebellar ataxia (SCA) [28]. It could be demonstrated in other SCA models that astrocyte reactivity often includes an activation of the NF-κB pathway and that silencing of this pathway by MyD88-knockout could rescue the astrocyte-induced neurodegeneration [29–32].

In MS, cerebellar GM pathology is not only apparent in demyelinated lesions [7, 13] but is further characterized by functional changes in connectivity and ion channel composition involving the NAGM [6, 33–36]. Thus, an abnormal repertoire of ion channels was described in dendrites of PCs of MS patients and chronic EAE mice, causing a disturbed electrical activity pattern in the cerebellum of EAE without evident neurodegeneration [33, 37, 38]. In EAE, those effects are mainly mediated by the pro-inflammatory cytokine IL-1β that is secreted by infiltrating leukocytes and activated microglial cells, thus causing an activation of the astroglial compartment. Similar to SCA, astrocytic reactivity causes downregulation of glutamate transporters and impaired glutamate uptake, leading to synaptic alterations and finally neurodegeneration [20]. Nevertheless, NAGM pathology is generally accepted to be a hallmark of chronic MS, where the effects of infiltrating leukocytes are negligible. Therefore, in the present study we used the cuprizone-induced toxic demyelination model to evaluate synaptic pathology in the cerebellar NAGM as an experimental paradigm mimicking key aspects of chronic MS.

## Results

### Cuprizone feeding induces astrogliosis and discrete microglia activation in the cerebellar grey matter in the absence of widespread cortical demyelination

**Fig. 1:**
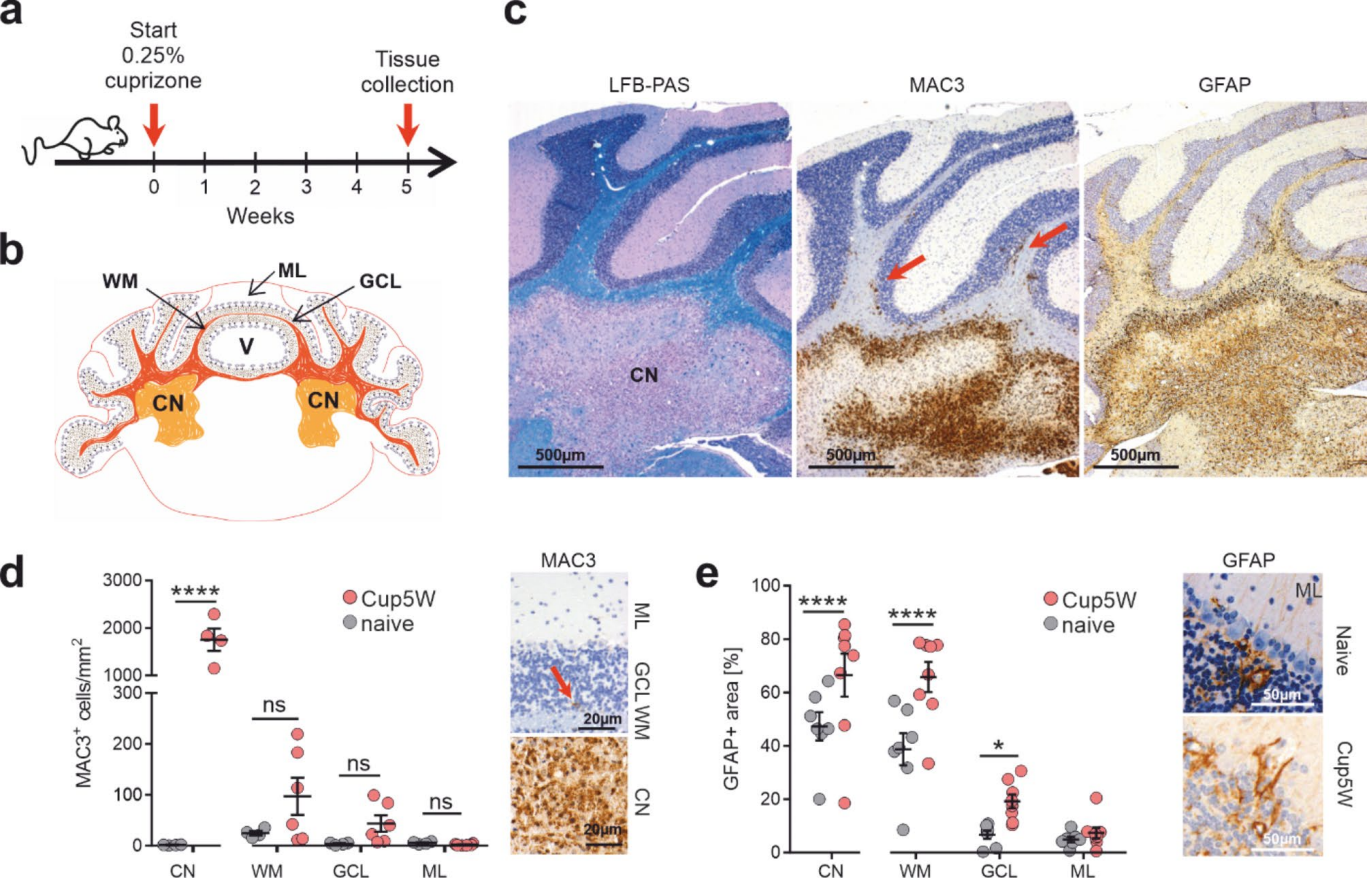
Cuprizone feeding induces demyelinated lesions with pronounced gliosis in the cerebellar nuclei with sparing of the cerebellar cortex. **a**. Cuprizone was administered for 5 consecutive weeks, followed by histological or electrophysiological analysis. **b** Schematic representation of the analyzed regions of the murine cerebellum, namely the cerebellar nuclei (CN) and the cerebellar cortex, consisting of the subcortical lobar white matter (WM), the granule cell layer (GCL) and the molecular layer (ML). Analysis of the cerebellar cortex was performed in the area of the vermis (v). **c** Representative overview micrographs depict the cuprizone-induced demyelinated lesion in the CN and the fully myelinated cortical region in LFB-PAS staining (left). Abundant MAC3^+^ activated microglial cells are detectable within the lesion. In the subcortical lobar WM and GCL close to the lesion, some activated microglial cells (red arrows) can be observed (middle image, MAC3 IHC). Astrogliosis is apparent in the lesion, to some extent reaching into the myelinated subcortical lobar WM and GCL close to the lesion (right image, GFAP-IHC). **d** Quantification of MAC3^+^ cells in different cerebellar regions, in and outside of the demyelinated lesion, comparing cuprizone-fed vs. naive mice. Higher magnification images (right upper panel) show the cerebellar cortex with only single MAC3^+^ cells (red arrow) in contrast to a massive microglial activation within the lesion (lower right panel). Each point represents a measurement from an individual animal (naive *n* = 4; CUP5W *n* = 6). **e** Quantification of GFAP^+^ area in different cerebellar regions of cuprizone-fed vs. naive mice. High magnification images (right panel) indicate GFAP^+^ Bergmann glia cells in naive (top) vs. cuprizone-fed mice (bottom) (GFAP-IHC). Each point represents a measurement from an individual animal (naive *n* = 7; wt CUP5W *n* = 8). Missing values in d (CN) correspond to insufficient cerebellar nuclei areas in two CUP5W samples. Whiskers represent mean ± SEM. P values were obtained after two-way ANOVA followed by Sidak’s multiple comparisons test. Asterisks represent significant p-values (*p < 0.05, **p < 0.01, ***p < 0.001, **** *p* < 0.0001)

To induce a demyelinating cerebellar pathology, wild type (WT) mice were fed with the copper chelator cuprizone for 5 weeks (Fig. 1a). Upon histological investigation, cuprizone-challenged mice showed pronounced progressive demyelinated lesions in the cerebellar white matter including the cerebellar nuclei, whereas the cerebellar cortex did not show any apparent myelin loss on LFB-PAS histochemistry (Fig. 1b, c). On the other hand, immunohistochemistry with proteolipid protein (PLP) showed a small but significant reduction in the subcortical lobar white matter and granule cell layer in cuprizone treated as compared to naive animals (*p* < 0.0001). However, these differences could not be confirmed in immunohistochemical analyses with the myelin proteins MBP or CNP (Supplementary Fig. 1). Cuprizone-fed animals showed abundant MAC-3-positive activated microglia within the demyelinating lesions in the region of the cerebellar nuclei (CN) as compared to naive animals (*p* < 0.0001) (Fig. 1d). In all three analyzed regions of the non-demyelinated cerebellar cortex (subcortical lobar white matter, granule cell layer, molecular layer) densities of MAC3-positive cells were low and no significant differences to naive control animals could be revealed (Fig. 1c, d). A similar distribution of area coverage was observed for Iba1 and TMEM119 immunohistochemistry, and no significant differences to naive animals were identified in any of the regions of the cerebellar cortex (Supplementary Fig. 1c, d). Moreover, cortical microglia showed a branched morphology in all conditions indicating no overt microglia activation in the cerebellar NAGM (Supplementary Fig. 1c, d). The expression of the early activation marker S100A9/MRP14 was restricted to sporadic cells with no significant differences in cell density between both conditions (Supplementary Fig. 1e).

It has previously been shown that T-lymphocyte infiltration does not play a prominent role in lesion formation and repair in the cuprizone model in the brain [39]. However, cerebellar demyelination is less well characterized, thus we quantified the density of CD3^+^ T-lymphocytes in the different cerebellar cortical regions as well as in the areas of demyelination in the cerebellar nuclei. Similar to the findings reported for the corpus callosum we observed a significant increase in CD3^+^ T-cells within demyelinated areas in the cerebellar nuclei (*p* < 0.0001) that did, however, not extend into the cerebellar cortex (Supplementary Fig. 1b).

Severe astrogliosis was observable within the lesion, indicated by an increased GFAP immunoreactivity (Fig. 1c, e). There was a significant regional increase in GFAP area coverage in cuprizone fed mice in the subcortical lobar white matter (WM, *p* < 0.0001) and granule cell layer (GCL, *p* < 0.05), where histopathologic investigation revealed reactive Bergmann glia that partially extended into the molecular layer (Fig. 1e, inset). Within the molecular layer, a slight increase in the GFAP-positive area corresponding to Bergmann glia processes was detectable, albeit not reaching statistical significance. Regional analysis of astroglia density using immunohistochemical labelling of Sox9 showed no significant increase in cell density in any of the cortical regions analyzed. In particular, we did not observe changes in cell density in the Purkinje cell layer, corresponding to Bergmann glia, in spite of increased GFAP expression (Supplementary Fig. 2b). Further, we analyzed the density of S100B positive cells in the vicinity of the Purkinje cell layer, since nuclear S100B expression in this region has been used as a marker of Bergmann glia [40, 41]. Here, we observed a trend for a reduction in cell density which failed to reach statistical significance (Supplementary Fig. 2a). Taken together our results show that cuprizone-induced demyelination was exclusively localized to the cerebellar nuclei and was accompanied by a pronounced local glial response in this region. Moreover, our results indicate that the cerebellar cortex in this model exhibits characteristic features of NAGM with increased reactive astrogliosis without overt microglial activation, demyelination or T cell infiltration.

### Relative structural neuro-axonal and synaptic integrity in the normal-appearing grey matter of cuprizone-fed mice

**Fig. 2:**
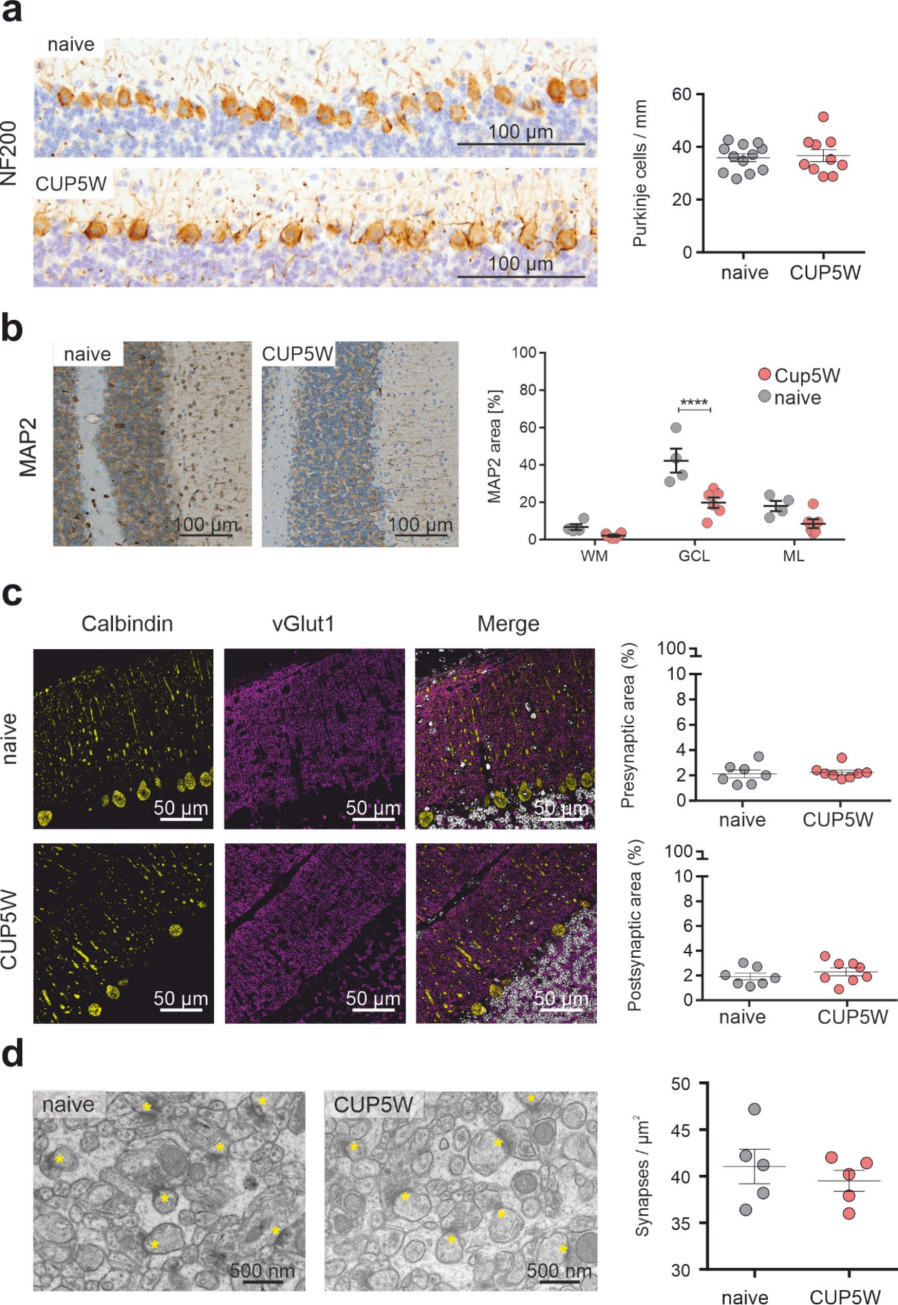
Cerebellar neuro-axonal and synaptic structural integrity in cuprizone-fed mice. **a** NF200-IHC of the Purkinje cell layer (left panel) and quantification (right panel) showing preserved PC numbers in naive and CUP5W. Purkinje cell numbers were normalized to the proximal perimeter of the molecular layer corresponding to the Purkinje cell layer. Each point represents average measurements from a single animal (naive *n* = 12; CUP5W *n* = 10). **b**. Representative images of MAP2-IHC and quantification showing a significant decrease in MAP2^+^-area in the granule cell layer (naive *n* = 4; CUP5W *n* = 6). **c**. Left panel: High magnification confocal images of the molecular layer fluorescently labelled with anti-calbindin (yellow) and anti-vGlut1 (magenta) antibodies. Nuclei are counterstained with DAPI (white). Right Panel: Quantification pre- (above) and postsynaptic area (below) as percentage of cerebellar cortex. Each point represents average values for a single animal (naive *n* = 7; CUP5W *n* = 8). **d**. Representative electron microscopic images of the molecular layer showing individual synapses (yellow asterisks) in naive and CUP5W mice (left panel), upon quantification (right panel) no significant differences in synaptic density could be observed. Each point represents average values for a single animal (naive *n* = 5; CUP5W *n* = 5). Whiskers represent mean ± SEM. P values were obtained after Mann Whitney test for a, c and d or two-way ANOVA with Sidak’s multiple comparisons for b. Asterisks represent significant p-values (*p < 0.05, **p < 0.01, ***p < 0.001, **** *p* < 0.0001)

Loss of dendrites is an important component of the primary pathology in the NAGM of MS patients in the brain [5]. We therefore performed a histochemical and immunohistochemical assessment of dendrite coverage. Immunohistochemical staining for MAP2 showed a significant reduction in the granule cell layer (*p* < 0.0001), and a trend towards a lower fiber coverage in the molecular layer of cuprizone-fed animals which failed to reach statistical significance (Fig. 2b). These differences, however, could not be observed in SMI31 immunohistochemistry (Supplementary Fig. 2d). We also determined dendrite crossings in Bielschowsky’s silver impregnation in the cerebellar cortex, and we observed a slight but significant neurite density reduction in the molecular layer (*p* < 0.05) of cuprizone-challenged animals but not in the other regions analyzed (Supplementary Fig. 3c).

Besides these subtle changes, further parameters indicative of neuro-axonal damage such as number of Purkinje cells (Fig. 2a), Purkinje cell spines, density of glomeruli and APP + spheroids did not show any significant alterations in cuprizone-treated animals (Supplementary Fig. 3). In particular, no significant reduction in v-Glut1 positive synapses was observed in the NAGM by confocal microscopy (Fig. 2c), which was confirmed at the ultrastructural level, where no significant differences in synaptic density were found (Fig. 2d).

### Differential expression of synaptic proteins in the cerebellar NAGM

**Fig. 3:**
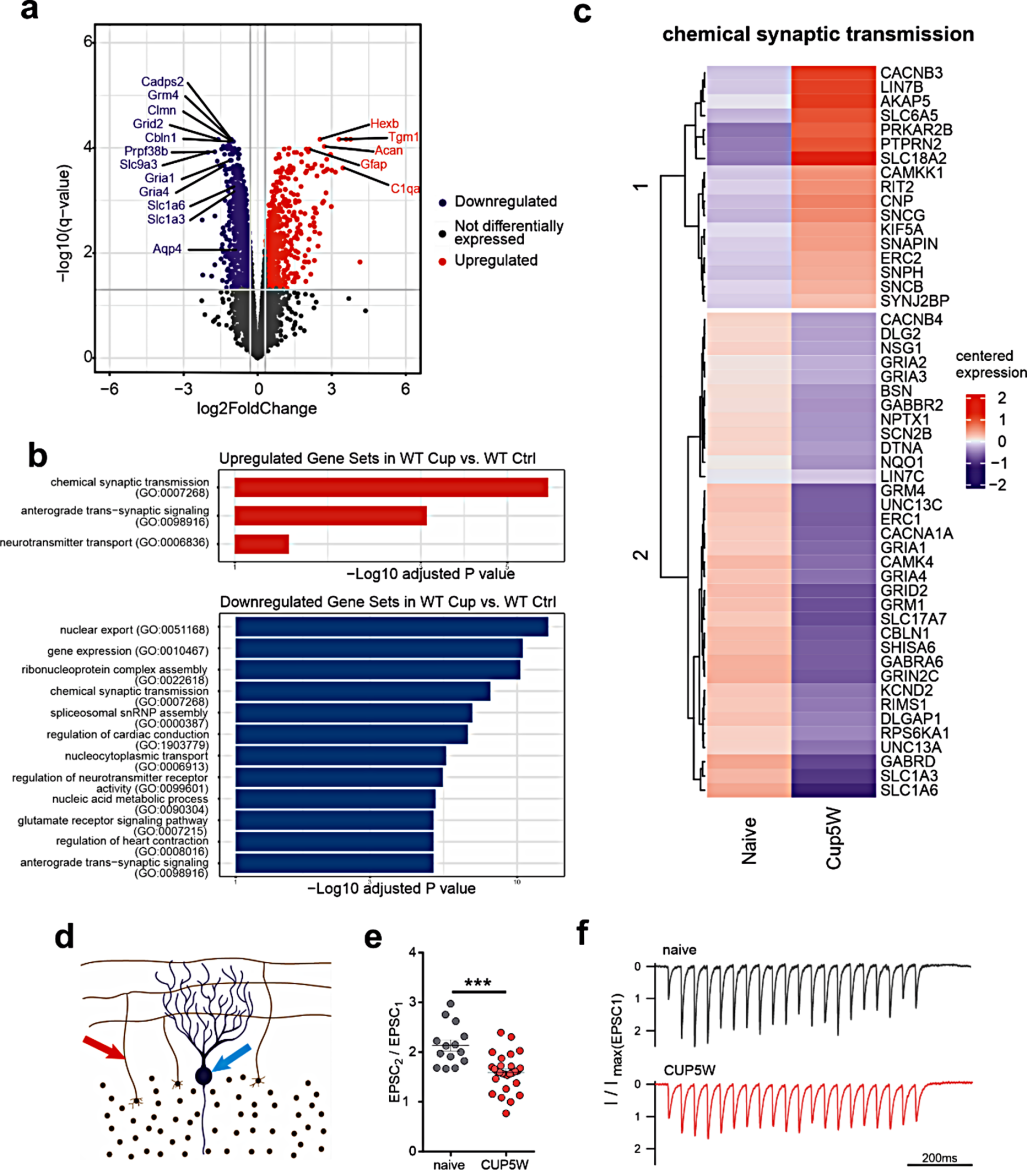
Differential expression of synaptic proteins in the cerebellar cortex and reduction of facilitation at the Purkinje cell - parallel fiber synapse in cuprizone-fed mice. **a** Volcano plot of proteomic analysis of the cerebellar cortex of wild type naive and cuprizone fed mice (CUP5W), depicting individual proteins and their regulation status in CUP5W as downregulated (blue), upregulated (red) and not differentially regulated (grey). Individual names are plotted for selected proteins (*n* = 3 independent samples per group). **b**. Up- (top) and down- (bottom) regulated gene sets from GO_Biological_process_2021 sets in WT mice after treatment with cuprizone. For clarity, gene sets containing the term “RNA” were filtered out, and up to 10 most significant gene sets are shown. The complete list can be found in supplementary Table 1. **c**. Heatmap representation of proteomic analysis showing differential expression of proteins related to glutamatergic synaptic transmission, showing an overall reduced relative expression in CUP5W. **d** Schematic representation of the PF-PC synapse. Parallel fibers are stimulated with an extracellular electrode (red) and EPSCs are recorded on PC using whole-cell patch clamp (blue arrow). **e** Relative amplitude of 2nd to 1st EPSC showing a reduction in CUP5W facilitation with time of cuprizone feeding. Each point represents an individual cell. Whiskers represent mean ± SEM, P values were obtained after unpaired t-test. Asterisks represent significant p-values (*p < 0.05, **p < 0.01, ***p < 0.001, **** *p* < 0.0001). f Representative excitatory post-synaptic current (EPSC) traces of naive (grey, top panel) and CUP5W (pink, bottom panel) animals upon stimulation of parallel fibers at 50 Hz

As no major morphological changes could be observed in the cerebellar cortex after 5 weeks of cuprizone feeding, we performed proteomic analysis as an unbiased approach to explore potential changes of the NAGM not identifiable on a morphological basis. Differential expression analysis identified 551 differentially regulated proteins, of which 223 were upregulated and 328 downregulated in cuprizone-treated wild type animals when compared to naive controls (Fig. 3a). The histologically observed reactive gliosis was reflected in the proteomic analysis by a ~ 2 fold increase in GFAP expression as well as differential expression of other astrocytic proteins such as Aquaporin 4 and the glutamate transporter EAAT1 (Slc1a3) (Fig. 3a, Supplementary Fig. 4). Gene set enrichment analysis of cerebella treated with cuprizone revealed that most downregulated gene sets (corresponding to the analyzed proteins) were those involved in mRNA processing and translation. The first gene set not representing RNA-related processes was “chemical synaptic transmission” which appeared both in up- and downregulated gene set lists (Fig. 3b). Gene sets involved in RNA processing were omitted from the display for clarity, a full list of gene sets and their respective p-values is given in Supplementary Table 1. Upon closer inspection, we found that a wide range of synaptic proteins was affected by treatment with cuprizone. Most notably, the glutamate transporters GLAST (EAAT1) and EAAT4 were strongly downregulated, followed by GRID2, AMPA- and ionotropic and metabotropic NMDA-receptor subunits. Also, SHISA6 which is important for tethering AMPA-receptors to the postsynaptic density was significantly reduced in animals treated with cuprizone. On the presynaptic side, expression of the vesicular glutamate transporter 1 (vGlut1, SLC17A7) was reduced as well as the voltage gated Ca2 + channel CACBA1A /Cav2.1) and the vesicle priming proteins MUNC13-a/-c and RIMS1 (Fig. 3c), indicating synaptic alterations in the NAGM. Based on the histological data, we also explored differences in protein distribution based on the cellular populations present in the NAGM, namely Bergmann glia and microglia. Differential expression analysis showed a pronounced up-regulation of GFAP and a reduction in proteins related to Bergmann glia such as AQP4, SLC1A3 (EAAT1) and S100B (Supplementary Fig. 4a). In line with the histological observations, only small differences in microglia activation genes were observed (Supplementary Fig. 4b).

### Synaptic facilitation is reduced at the glutamatergic parallel fiber Purkinje cell synapse (PF-PC) in cuprizone-fed animals

To assess the functional effects of the differential expression of synaptic proteins and in particular of glutamatergic transmission in the NAGM we performed electrophysiological measurements at the glutamatergic parallel fiber Purkinje cell synapse (PF-PC) (Fig. 3d). As expected for this synapse, in naive animals a frequency-dependent increase in the amplitude of PC excitatory post-synaptic currents (EPSC), from first to the second EPSC (EPSC_2_/EPSC_1_), of about 1.6, 1.93 and 2.14-fold was observed with extracellular stimulation of granule cells at frequencies of 10, 20 and 50 Hz, respectively (Supplementary Fig. 5). In contrast, 5-week cuprizone-fed mice showed a significantly reduced facilitation at 50 Hz (*p* < 0.001) (Fig. 3e, f). A reduced facilitation was also observed at 10 and 20 Hz stimulation frequencies yet it was slightly less pronounced and failed to reach statistical significance (Supplementary Fig. 5). No significant differences were observed in the decay time constant of the EPSCs between naive and cuprizone-fed animals (Supplementary Fig. 5).

### MyD88 deficiency modulates synaptic protein expression and associates with restored PF-PC facilitation in cuprizone treated animals

**Fig. 4:**
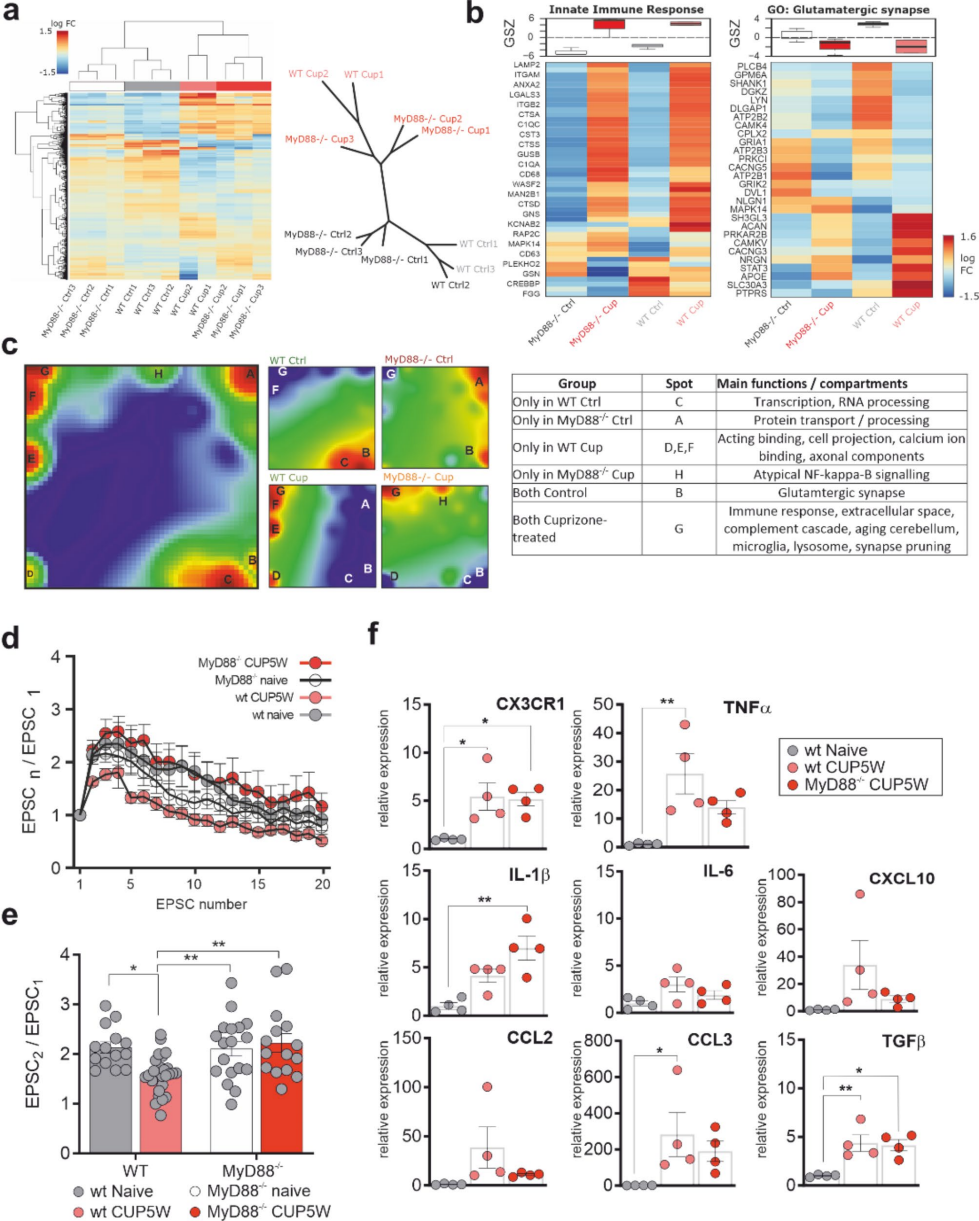
Differential regulation of synaptic proteins and inflammatory cytokines in cuprizone treated MyD88 deficient mice. **a** Pairwise sample correlation (left panel) and phylogenetic distance clustering (right panel) of samples based on protein expression in the cerebellar cortex of wild type (WT, gray and pink) and MyD88^−/−^ (white and red) mice with (Cup) or without (Ctrl) 5 week cuprizone feeding (*n* = 3 independent samples per group). Note that cuprizone treatment had a stronger effect than genotype in sample correlation and clustering. **b** Heatmap representation and Gene Set Z-score (GSZ) of proteome analysis revealing differential expression of proteins related to innate immune system response (reactome, left panel) and glutamatergic synapse (gene ontology, right panel). **c** Self organizing maps (SOM) and sample portraits for the different experimental conditions depicting overexpression spots (A-G) and their specific expression and function in different groups (table). Normalized Purkinje cell EPSC amplitudes at 50 Hz stimulation frequency of parallel fibers presented as a stimulus train (**d**) or 2nd EPSC (**e**) show a reduction in facilitation in WT animals after cuprizone treatment (wt CUP5W, pink) as compared to naive WT animals (wt naive, grey). Each point represents mean normalized EPSC value ± SEM. No differences in facilitation are observed in naive (MyD88^−/−^ naive, white) or cuprizone fed (MyD88^−/−^ CUP5W, red). Each point represents an individual cell. Bars’ heights represent mean ± SEM. **f** Quantification of relative mRNA expression (qPCR) of selected immune modulatory mediators in the cerebellar nuclei (CN) showing a significant increase in several genes (CX3CR1, CCL3, TNFα, and TGFβ) in WT animals after cuprizone treatment. A similar cytokine upregulation of CX3CR1 and TGFβ was observed in MyD88^−/−^ mice who also showed an increase in IL-1β and a trend towards a decrease in TNFα and CCL3 when compared to WT CUP5W mice. Each point represents mean relative expression of two technical replicates for a single animal. Bar height represents mean relative expression ± SEM. P values were obtained after two-way ANOVA followed by Tukey’s multiple comparisons test (**e**) and one-way ANOVA followed by Tukey’s multiple comparisons test (**f**). Asterisks represent significant p-values (*p < 0.05, **p < 0.01, ***p < 0.001, **** *p* < 0.0001)

MyD88 is an adaptor protein involved in TLR/IL-1R-mediated signaling central to the innate immune response and likely involved in cerebellar synaptic pathology. For instance, in EAE distant functional synaptic changes are mostly induced by IL-1β and likely mediated by activation of microglia and T-lymphocyte infiltration [20, 42, 43]. Also, MyD88-deficiency has been shown to rescue astrocyte-induced neurodegeneration in models of cerebellar neurodegeneration [29–32]. Therefore, to gain a deeper mechanistic insight into our model characterized by a paucity of microglial activation and T-lymphocyte infiltration in the NAGM we explored the potential influence of the innate immune system activation on NAGM synaptic pathology in animals deficient in MyD88. In order to exclude differences in the basic cellular composition and response to cuprizone feeding between MyD88^−/−^ and wildtype animals we performed a detailed histological characterization. We did not observe any differences in myelination status, dendrite density, astrocyte coverage, Bergmann glia density or microglia/macrophage area and activation in the NAGM between wildtype and MyD88^−/−^ naive mice and after 5-week cuprizone feeding (Supplementary Figs. 1, 2 and 4). Further, we evaluated the overall composition of the inflammatory milieu in cuprizone-induced demyelination in wildtype and MyD88^−/−^ animals by measuring the relative expression of genes related to innate immunity in the white matter lesion areas in the cerebellar nuclei. An increase in the relative expression of TGFβ and CX3CR1 was observed after 5 weeks of cuprizone feeding in wildtype and MyD88^−/−^ animals as compared to WT naive mice. For TNFα a significant increase was observed only in cuprizone-fed WT animals while MyD88^−/−^ CUP5W showed intermediate TNFα levels between naïve and WT CUP5W which failed to reach statistical significance. IL-1β showed an increase in relative expression in MyD88^−/−^ CUP5W only, and no differences were observed in CCL2, CCL3, IL4 and CXCL10 between any of the groups (Fig. 4f).

We then analyzed the proteome of the cerebellar cortex of MyD88^−/−^ and wild type (WT) mice with (CUP) or without (Ctrl) cuprizone treatment. Interestingly, phylogenetic distance clustering of samples based on protein expression data as well as pairwise sample correlation revealed a stronger effect of cuprizone treatment than genotype (Fig. 4a, b), suggesting a stereotypical response to demyelination (Fig. 4b) that was either independent of MyD88 or that can be carried out through alternative pathways. This is in line with our histological analyses showing a similar extent of demyelination and microglial activation in MyD88^−/−^ and wild type animals (Supplementary Fig. 1 and supplementary Fig. 2).

By using self-organizing map (SOM) portrayal we could identify overall seven spot-like modules of co-regulated proteins (labeled with capital letters A-H) (Fig. 4c). Moreover, specific activation or deactivation of protein modules for each condition was determined by means of condition-averaged SOM portraits (Fig. 4c). Further highlighting the similarities in the effects of cuprizone feeding, both genotypes showed a strong activation of elements of the immune system, the extracellular matrix and microglia (Spot G) and a downregulation of proteins involved in glutamatergic synaptic transmission and RNA processing (spot B and C) (Fig. 4b, c). In cuprizone-treated wild type mice we observed an upregulation of spots D, E, and F, which comprise components of the cytoskeleton while spot H, containing components of the atypical NF-κB pathway, a transcription factor downstream of MyD88, was exclusively active in cuprizone-treated MyD88^−/−^ mice (Fig. 4c).

Differences between groups were analyzed by subtracting condition-averaged SOM-portraits from each other. While spots D, E, F, and G (oligodendrocyte markers, cell projections, extracellular region, immune system) are upregulated in wild type NAGM after cuprizone treatment, spots B and C, which contain pre- and postsynaptic proteins as well as RNA-processing elements, are downregulated. In MyD88^-/-^ mice, cuprizone treatment had a lesser effect on protein expression. Only 73 proteins were significantly up- or downregulated. Of these, proteins in spot G (immune system) were upregulated after cuprizone treatment, while spot B (a subset of synaptic proteins) and a subset of spot A and D (endoplasmatic reticulum, oligodendrocyte markers) were downregulated. Proteins related to cell projection, neural crest, stem cell and oligodendrocyte markers, represented in spots E and D, were also less expressed in MyD88 deficient mice. On the other hand, MyD88^-/-^ mice showed an upregulation of proteins in dispersed SOM-regions and in proteins related to atypical NF-κB pathway (spot H).

**Fig. 5:**
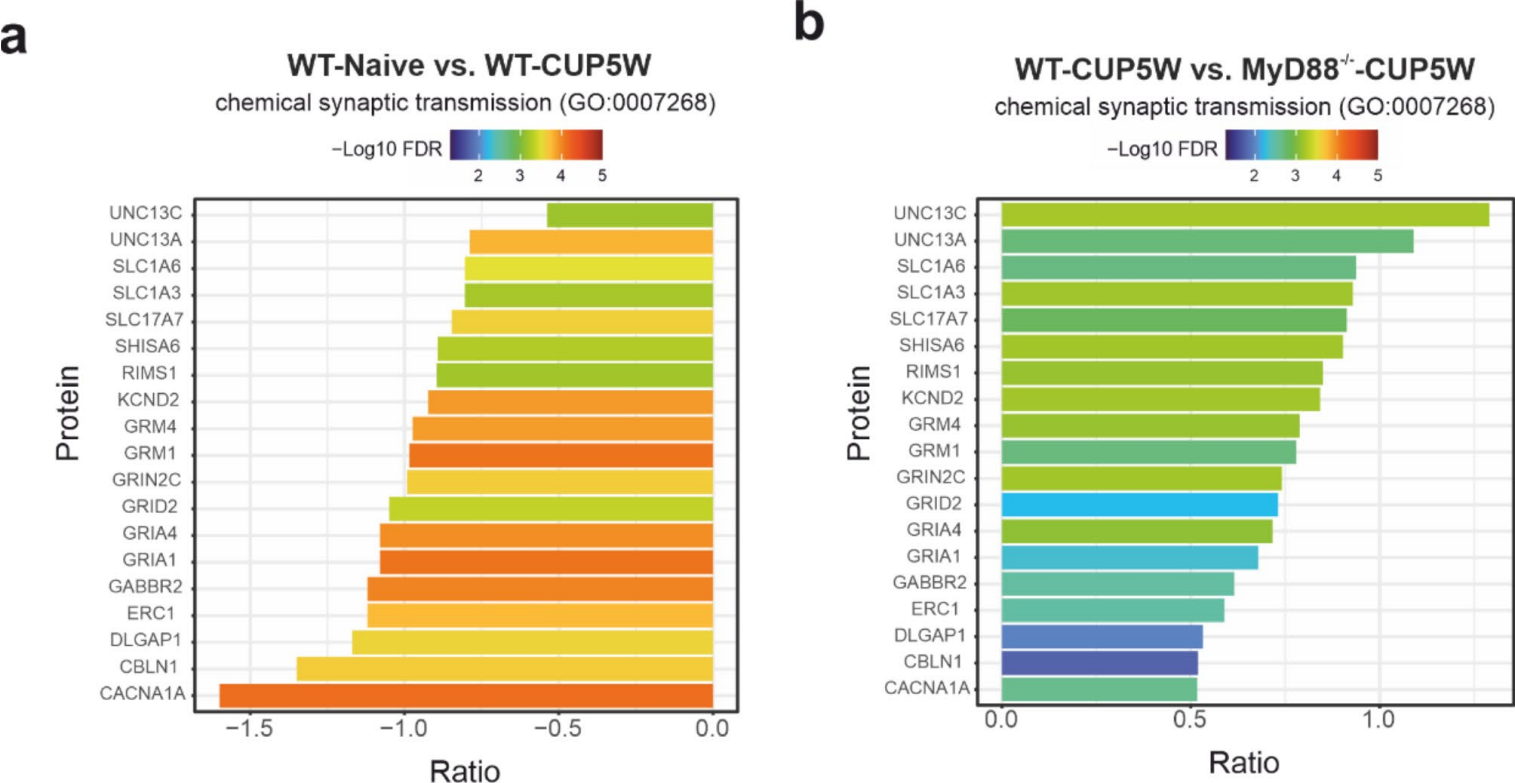
Rescue of expression of synaptic proteins in MyD88^-/-^ mice. Cuprizone treatment reduced the expression of a number of genes in the “chemical synaptic transmission” gene set (see also Fig. 3). In MyD88^-/-^ animals, a substantial subset of these genes were not or only slightly altered upon cuprizone treatment. **(a)** Ratios of expression after and before cuprizone treatment in WT animals. **(b)** Ratio of expression between MyD88^-/^ and WT animals treated with cuprizone. The color corresponds to the -log10 transformed FDR. Most prominently, the glutamate transporters SLC1A6 and SLC1A3 (EAAT4 and GLAST), proteins involved in synapse structure and maintenance (e.g. GRID2, CBLN1, SHISA6), and several glutamate receptor subunits (GRIN2C, GRM1, GRM4, GRIA1, GRIA4) are rescued

Taken together, MyD88 deficiency was associated with a rescue of the levels of glutamate transporters and several components of the postsynapse, including SHISA6 and DLGAP1 (GKAP), which are responsible for tethering AMPA receptors at the postsynaptic density (PSD), AMPA- and NMDA-receptors, and the CLBN1-GRID2-complex. Further, expression of some presynaptic factors governing neurotransmitter release is increased in the MyD88^-/-^ with cuprizone in relation to WT with cuprizone (Fig. 5 and Supplementary Fig. 6).

In particular, a differential regulation of the astrocytic glutamate transporters GLAST/EAAT1 and EAAT4 has been implicated in synaptic dysfunction in models of inflammatory demyelination [40, 41]. In line with the proteomic data, we observed a reduction in EAAT1/GLAST area in the molecular layer of cuprizone-fed wildtype animals (*p* < 0.001) compared to naive wildtype and both MyD88^-/-^ naive and cuprizone-fed mice (Supplementary Fig. 3). Moreover, the differences in the composition of glutamatergic synaptic proteins between wildtype and MyD88^-/-^ (Fig. 4b) were associated with distinct functional synaptic phenotypes, whereby no significant differences in facilitation at the PF-PC synapse in MyD88^-/-^ mice were detected after 5-week cuprizone feeding (Fig. 4e).

## Discussion

Synaptic alterations are a consistent feature of NAGM pathology in MS and experimental models. The specific spatial-temporal synaptic phenotype depends on the interaction of different cellular populations and their humoral milieu, intrinsic synaptic characteristics and adaptive mechanisms (reviewed in [43]), thus reflecting lesion stage and distinct pathophysiology. Probably the best-studied molecular mechanisms leading to cerebellar synaptic pathology in the NAGM have been described in EAE and involve infiltration of T-lymphocytes and related inflammatory mediators, thus mimicking early active MS [20, 40–42]. For chronic MS, on the other hand, the major determinants are the continuous activation of the innate immune system and progressive NAGM pathology [44], for which the underlying cellular and molecular mechanisms are largely unexplored.

In the cuprizone model, changes in ion channel composition [45, 46], synaptic proteins [47, 48] and local synaptic loss [48] have been associated with demyelination in several brain regions [45–54]. In line with these results, we have previously described synaptic loss in the cerebellar nuclei in MS [7]. In the present study we cover new ground by specifically analyzing the cerebellar NAGM, where besides a discreet reduction in dendritic density in the molecular and granular layer we neither observe synaptic loss nor changes in spine density of Purkinje cells.

Nevertheless, in spite of relative structural synaptic integrity, we report here a differential expression of synaptic proteins involved in glutamatergic transmission associating with a functional phenotype namely a reduction in facilitation at the glutamatergic PF-PC synapse. While a reduction in MUNC13 and/or RIMS1 leads often to an increase in facilitation [55], the reduced expression of the voltage gated Ca^2+^ channel Cav2.1 might counteract this effect [56]. Further, the loss of glutamate transporters can lead to an increase in glutamate in the synaptic cleft and subsequent postsynaptic damage and receptor saturation. Additionally, ionotropic glutamate receptor subunits are reduced in animals treated with cuprizone. Several components important for synapse maintenance and stability are downregulated in response to cuprizone, including CLBN1, which together with GluRð2 (GRID2) and Neurexin forms a trans-synaptic complex for synapse formation [57]. Moreover, the reduction in astrocytic glutamate transporters is likely to contribute to increased glutamate availability further compromising physiological synaptic function.

Interestingly, facilitation is restored in MyD88^−/−^ mice treated with cuprizone. This offers the possibility to compare components of the synaptic machinery to identify the factors contributing to the reduced facilitation. We observe a rescue of the glutamate transporters and several components of the postsynapse, including SHISA6 and DLGAP1 (GKAP), which are responsible for tethering AMPA receptors at the PSD, AMPA- and NMDA-receptors, and the CLBN1-GluRð2-complex. Further, expression of some presynaptic factors governing release is increased in MyD88^−/−^ animals fed with cuprizone with regard to WT with cuprizone. The glutamate transporters also show similar levels as compared to untreated controls, thus likely providing a more stable glutamate homeostasis. Altogether, the interdependence of the above mentioned physiological pathways suggest that the mechanism underlying the reduced facilitation is likely the result of multifactorial molecular processes.

By performing recordings of this particular synapse – with physiologically unmyelinated axons [58–61] – we aimed to exclude the effects of local demyelination on action potential generation / propagation, network activity and synaptic transmission [45–47, 51–53, 62–65].Therefore, the synaptic changes in the cerebellar NAGM in our model point towards pathophysiological factors other than myelination status which are so far poorly understood in the cuprizone model [51].

For instance, afferent axonal transection and neuronal loss are known to contribute to synaptic alterations in MS and EAE [2, 6–8, 66–71]. However, the cuprizone model is not characterized by extensive neuronal loss under standard experimental protocols (reviewed in [72–75]), likely owing to the immunological features accompanying demyelination in this model. Nevertheless, we could observe a small but significant reduction in fibers in two regions of the NAGM layer. However, neurite loss was not consistent across the methods used since we observed a fiber reduction in the molecular layer in Bielschowsky’s silver impregnation, while MAP2–IHC revealed a fiber reduction in the granule cell layer. This variability might reflect regional differences in the sensitivity of each method and suggests that neurite loss in our model is around the limit of detection of histological methods.

A further factor contributing to synaptic pathology in inflammatory models and MS is the interplay between T-lymphocytes, microglia, astrogliosis and inflammatory milieu [14, 20, 39, 42, 43, 76–83]. One of the principal mechanisms described points towards overall increased glutamate availability, likely related to deficient modulation of glutamate uptake and release in different cellular populations (reviewed in [83]). In this study, however, we focused on the NAGM and show a paucity of microglial activation and no infiltration of T cells, thus allowing us to narrow down the contributions of the individual cellular populations involved. Nevertheless, we found a downregulation of glutamate transport proteins (GLAST and EAAT4) in WT but not MyD88^−/−^ cuprizone-treated mice, thus suggesting a role for astrocytes/Bergmann glia in synaptic pathology in the NAGM in our model. Therefore, our data suggests glutamate dysregulation as a converging mechanism of synaptic pathology in inflammatory and toxic demyelination models, albeit with differences in the initial pathogenic events.

Of note, we could observe a significant increase in TNF-alpha and CCL3, which was less prominent in MyD88-deficient animals and in turn was associated with partially restored levels of glutamate transporters/synaptic proteins and an increase in facilitation at the PF-PC synapse relative to wild type mice. In contrast, the levels of IL-1β were further increased in MyD88^−/−^ upon cuprizone treatment, despite an amelioration of the synaptic phenotype, suggesting a less prominent role for this cytokine in synaptic pathology in our particular experimental paradigm as compared to previous published work in inflammatory models [43].

Altogether, our work highlights the complex regulation of synaptic transmission in demyelinating disorders, specifically in the cerebellar NAGM, and emphasizes the role of astroglia and in particular Bergmann glia activation, as key cellular determinants of cerebellar synaptic pathology. Furthermore, our results support glutamate dysregulation as a common mechanism for synaptic pathology and show complex synaptic changes associated with synaptic pathology (Supplementary Fig. 6).

Moreover, our work validates the cuprizone model as a tool for the study of primary synaptic changes independent of T cell infiltration, thus bringing forward an experimental paradigm mimicking key aspects of NAGM pathology in late-stage MS.

## Materials and methods

### Animals

Transgenic MyD88^−/−^ mice were obtained from the animal facility at the University of Göttingen or from The Jackson Laboratory (Bar Harbor, ME, IMSR_JAX:009088). Wild-type (WT) C57BL/6 mice were obtained from Charles River Laboratories (Sulzfeld, Germany). All animal experimentation was carried out in accordance with the European Council Directive. Experiments were approved by the Lower Saxony Federal State Office for Consumer Protection and Food Safety, Germany.

### Cuprizone treatment

Toxic demyelination was induced by cuprizone (oxalic bis[cyclohexylidenehydrazide]; C9012, Sigma-Aldrich, Germany). Ten-week-old WT and MyD88^−/−^ mice received a cuprizone (0.25%) diet *ad libitum* mixed in ground chow in our premises as described in previous studies from our group [84–87] for 1, 3 or 5 weeks to study synaptic alterations of the normal appearing cerebellar cortex. Body weights of mice were controlled once weekly. Mice fed with standard rodent chow were used as naive controls.

### Preparation of acute cerebellar slices

After 5 weeks, WT and MyD88^−/−^ animals were anesthetized with isoflurane and immediately decapitated. Preparation of acute cerebellar slices was carried out as described elsewhere [88]. Briefly, the cerebellum was isolated in ice-cooled oxygenated artificial CSF (ACSF) containing (in mM): 60 NaCl, 120 sucrose, 25 NaHCO_3_, 1.25 NaH_2_PO_4_, 2.5 KCl, 25 D-glucose, 0.1 CaCl_2_, 3 MgCl_2_, 3 myo-inositol, 2 sodium pyruvate and 0.4 ascorbic acid. 200 μm thick coronal vibratome slices of the cerebellar vermis were cut (VT1200S; Leica, Wetzlar, Germany) and then transferred to oxygenated ACSF (containing in mM: 25 NaCl, 2.5 KCl, 25 NaHCO_3_, 1.25 NaH_2_PO4, 2 CaCl_2_, 1 MgCl_2_, 3 myo-inositol, 2 sodium pyruvate and 0.4 ascorbic acid) at a temperature of 36 °C for 45 min to 1 h.

### Electrophysiological recordings

Recording was done in temperature controlled (37 °C) and oxygenated ACSF. Purkinje cells were visualized using differential interference contrast microscopy. For extracellular stimulation of the granule cell layer, an electrode was placed in the granule layer and the stimulation intensity was adapted to elicit a Purkinje-cell excitatory postsynaptic current (EPSC) of 100 pA. Patch pipettes were pulled to a resistance of 3-4MΩ using WPI.PG10165-4 glass (World Precision Instruments) and a L/M-3P vertical puller (List Medical) and filled with intracellular solution containing (in mM): 135 potassium gluconate, 5 KCl, 10 Hepes, 5 MgATP, 0.5 NaGTP, 1 EGTA and 5 *N*-(2,6-dimethylphenylcarbamoylmethyl)-triethylammonium chloride (QX-314; Tocris, Ellisville, MO, USA). For recording, a holding potential of -60mV was applied and stimulation frequencies of 10, 20 and 50 Hz were used. Currents were sampled at 50 khz and low-pass filtered at with a 2.9khz Bessel filter. Data were acquired with an EPC10/2 amplifier (HEKA) controlled by Patchmaster (v. 2.20, HEKA). Data analysis was performed using Igor Pro (Wavemetrics) as previously described [89].

### Histology and imaging

To analyze cuprizone-induced cerebellar pathology, mice were transcardially perfused with 4% paraformaldehyde after 1, 3 or 5 weeks of cuprizone treatment. Tissue asservation was carried out according to standard protocols, and paraffin-embedded tissue was used for further analysis. Paraffin sections were conventionally stained with Luxol fast blue-periodic acid-Schiff (LFB-PAS) to assess cerebellar demyelination, and Bielschowsky’s silver impregnation to study neurite densities as described elsewhere [90, 91]. For visualization of dendritic spines, tissue slides were stained using the Golgi-Cox method as previously described [5]. For the quantification of dendritic spines the number of spines per dendrite length (µm) was used as the unit of measurement. A minimum of 60 μm were measured in each animal. For immunohistochemical analysis, the following primary antibodies were used: glial fibrillary acidic protein (GFAP, 1:1000, Dako Z0334), activated microglia (MAC3, 1:200, BD Pharmingen Clone M3/84), Synaptophysin (1:50, Dako A0010) and neurofilament (NF200, Clone N52, 1:400, Sigma Aldrich N0142), S100 beta (1:100, Abcam ab7853-500), EAAT1 (1:100 Alpha Diagnostic, GLAST-11 A,), CNP (1:200, Biolegend, SMI-91), PLP (1:500; Biorad, clone plpc1), Iba1 (1:100, Merck, MAB N92), TMEM119 (1:100, Abcam, clone 106-6; ab220249) SMI31/NF-H (1:10000, Merck Millipore, clone SMI31), S100A9 (rabbit polyclonal, kindly provided by C. Sorg, Münster, Germany) (Nogai et al. 2005), myelin basic protein (MBP, Cellmarque 295 A-16), microtubule associated protein 2 (MAP2, Sigma M4403, Clone HM-2), SRY-Box Transcription Factor 9 (Sox9, ThermoScientific MA5-41174, Clone SN74-09). Biotinylated secondary antibodies (GE Healthcare, Jackson ImmunoResearch and DCS Innovative diagnostic system), peroxidase-conjugated avidin and DAB (Sigma Aldrich) were used for immunohistochemistry. Immunofluorescence double-labeling of the synaptic vesicular markers (vGlut1, 1:100, Synaptic Systems 135302; Calbindin 1:100 (D28K) 214005 Synaptic systems) was performed using fluorescence labeled secondary antibodies. For automated immunohistochemistry of MBP, MAP2, Sox9 and GFAP the Benchmark Ultra Staining System was used. Microphotographic scans of stained tissue sections were acquired with a VS120 Virtual slide microscope (Olympus) using the cellSense Dimension software (Olympus). Cerebellar regions were manually delineated for analysis using ImageJ (FIJI) [92] and QuPath (version 0.5) software [93]. Cell densities were determined by manual counting of cells using the ImageJ cell counter plugin or by automatic intensity-based DAB detection with posterior manual control. The obtained cell numbers were divided by the respective area and are given as cells/mm^2^. For determination of Purkinje cell numbers, the absolute number of Purkinje cells was normalized to the proximal perimeter of the molecular layer corresponding to the Purkinje cell layer. A diameter of 70 μm around the aforementioned perimeter was used as the normalization area for S100B positive cell density. For quantification of white matter lesion areas, the region of the cerebellar nuclei was selected (CN). Then the region of interest (ROI) was expanded by 100 μm to include the lesion border. Expansion areas into the granule cell layer, brainstem or outside the tissue sections were excluded from the analysis. For quantification of area coverage measurements the threshold was set at one standard deviation above mean DAB background intensity as individually determined for each image. Areas with sectioning or border artifacts were excluded from the analysis. For detection of low density events (< 15 cells /mm^2^) the whole area of the vermis and or cerebellar nuclei was screened with the fast cell count plugin in QuPath software followed by visual confirmation and manual selection of the detected events. Subregion areas (CN, WM, GCL or ML) for calculation of cell density were only recorded in areas harboring positive events. Otherwise, cell density was assigned a value of 0 cells/mm^2^. Axonal densities determined using Bielschowsky’s silver impregnation were measured using an axonal counting grid with 25 cross-points, where the number of axons intersecting with the crossing points was determined as a fraction of the total number of cross-points at a magnification of ×400 as previously described [94]. The tight packing of axons in the cortical white matter prevented a reliable quantification using this method and was therefore excluded from the analysis.

Statistical analysis of two groups was performed using student’s t-test or Mann Whitney U as appropriate. One- or two-way ANOVA with Tukey’s or Sidak’s multiple comparisons was used for the analyses of several groups or two factors respectively. The specific tests and parameters used are mentioned in the respective section in the text or figure legends.

For characterization of synaptic contacts in the cerebellar cortex, fluorescence signals were collected with a Leica TCS SP8 X LIGHTNING FALCON confocal microscope (Leica) and the Leica LAS X software (Leica). Post-acquisition analysis was performed with ImageJ [95]. In short, 4–6 z-stacks of the cerebellar vermis were performed per animal. One image per stack was selected for further analysis based on the highest overall intensity profile in the calbindin channel. The molecular layer was manually defined as a region of interest (ROI). Background subtraction was performed for calbindin and vGlut1 separately by subtracting the mean value of a manually selected background area. The threshold for each channel was then automatically determined as the mean value in the background area + 2.5 standard deviations. Pre, post and colocalizing areas were measured and normalized to cortex area. For statistical analysis the mean value of all images for one animal were pooled.

### Electron microscopy

Tissue preparation and electron microscopy (EM) were carried out according to Weil et al. [95]. In brief, mice were killed by an overdose of Avertin before transcardial perfusion. After initial flushing with HBSS, mice perfused with 4% PFA, 2.5% glutaraldehyde in phosphate buffer containing 0.5% NaCl, pH 7.4 and fixed overnight at 4 °C. The Cerebellar vermis was sliced in sagittal direction using a 1200 S vibratome (Leica Microsystems, Wetzlar, Germany) and embedded in EPON (Serva, Heidelberg, Germany) after post-fixation with 2% OsO_4_ (Science Services, Munich, Germany) in 0.1 M phosphate buffer pH 7.3 and acetone dehydration. Ultrathin sections of the molecular layer of lobe V were prepared with a Leica UC7 ultramicrotome (Leica Microsystems, Wetzlar, Germany) using a 35° diamond knife (Diatome, Biel, Switzerland) and then stained using UranylLess™ (Science Services, Munich, Germany). EM pictures were obtained with a Zeiss LEO912 electron microscope (Carl Zeiss Microscopy GmbH, Oberkochen, Germany) equipped with a on-axis 2k CCD camera (TRS, Moorenweis, Germany). Twelve images per mouse were taken at a magnification of 16.000x. The AnalySIS image processing software 3.2 was used to calculate total number of synapses and vesicle numbers.

### qPCR

To assess relative mRNA expression levels, qPCR of macroscopically isolated cerebellar nuclei was performed. Total RNA was isolated from fresh brain tissue using the RNeasy Micro Kit (Qiagen) and mRNA was transcribed into cDNA using the High-Capacity RNA-to-cDNA™ Kit (Life Technologies) according to manufacturer’s instructions. Further, cDNA was used for qPCR using the qPCR core kit (Eurogentec). The following TaqManTM primers were obtained from Thermo Fisher Scientific (USA) and used as indicated by manufacturer’s protocol: Mm00441242_m1 (CCL2), Mm00441259_g1 (CCL3), Mm02620111_s1 (CX3CR1), Mm00445235_m1 (CXCL10), Mm00439620_m1 (IL-1 α), Mm00434228_m1 (IL-1β), Mm00446190_m1 (IL-6), Mm01178820_m1 (TGFβ), Mm00443258_m1 (TNFα). Analysis of qPCR was perfomed as previously described [96]. In short, for each biological sample (*n* = 4 per group) two technical replicates were used. The arithmetic mean of the CT values for every duplicate was taken as representative for the respective biological sample. Each mean CT value was linearized for normalization of target genes using the 2-ΔΔCt method [97, 98]. Glyceraldehyde-3-phosphate dehydrogenase (Gapdh) was used as a housekeeping gene. The wildtype naive group was used as experimental calibrator.

### Proteomic analysis

Proteomic analysis was performed on acutely isolated cerebellar vermis from naive (*n* = 3) and 5-week cuprizone-fed (*n* = 3) wild-type and MyD88^−/−^ animals. Each independent sample corresponded to the vermis from a single mouse. Tissue processing and Liquid-Chromatography Mass Spectrometry analysis were performed as previously described [99]. Hundred micrograms of tissue samples were taken for digestion. Samples were processed using a modified FASP protocol. The reduction was done using Biognosys’ Reduction Solution for 30 min at 37 °C and Alkylation was carried out using Biognosys’ Alkylation Solution for 30 min at room temperature in the dark. Subsequently, digestion was carried out overnight at 37 °C using trypsin (Promega) at the protein: protease ratio of 100:1. All the steps were performed on VIVACON 500 Membranes (Sartorius) with 30’000 Da MWCO. Peptides were desalted using C18 MacroSpin columns (NestGroupInc.) according to the manufacturer’s. instructions and dried down using a SpeedVac system. Peptides were resuspended to the theoretical peptide concentration of 2 µg/µl in LC solventA (1%acetonitrile, 0.1%formicacid (FA)). Peptide concentrations were measured using a UV/VIS spectrometer (SPECTROstarNano, BMGLabtech).

For the LC-MS/MSHRM measurements, 2 µg of peptides were injected to an in-house packed C18 column (Dr.MaischReproSilPur, 1.9 μm particle size, 120Å pore size; 75 μm inner diameter, 50 cm length, NewObjective) on a ThermoScientific EasynLC1200 nano-liquid chromatography system connected to a ThermoScientific QExactive HF mass spectrometer equipped with a standard nano-electrospray source. LC solvents were A:1% acetonitrile in water with 0.1% FA; B:15% water in acetonitrile with 0.1% FA. The non linear LC gradient was 1–55% solvent B in 120 min followed by 55–90% B in 10 s and 90% B for 10 min. For HRM on the QExactive HF a DIAmethod with one fullrange survey scan and 22DIA windows was used, the total gradient length was 135 min.

HRM mass spectrometric data were analyzed using Spectronaut software (Biognosys). The false discovery rate on peptide and protein level was set to 1%. Data was filtered using row based extraction. Data quality for mouse samples was analyzed using Biognosys’ Spectral Library Online Repository (Spectronaut 8, mouseliver). The normalization applied in Spectronaut was the local regression normalization [100]. The heatmap was generated using heatmap.2 of the ggplot2 package in the statistical package R. Distance was calculated using the “manhattan” method, the clustering using “ward.D”. For subsequent analysis, we applied oposSOM software [101–103]. Gene Set Z-Scores (GSZ) were calculated as described elsewhere [104].

## Electronic supplementary material

Below is the link to the electronic supplementary material.

Supplementary Material 1

Supplementary Material 2

## Data Availability

The datasets used and/or analyzed during the current study are available from the corresponding authors on reasonable request. Proteome data that support our findings are deposited on a publicly available file server.
