# Supplementary material for: Astroglial modulation of synaptic function in the non-demyelinated cerebellar cortex is dependent on MyD88 signaling in a model of toxic demyelination
Source: J Neuroinflammation. 2025 Feb 23;22:47. doi: 10.1186/s12974-025-03368-9 (PMC11849172; doi:10.1186/s12974-025-03368-9)
Supplement: Supplementary file 1 — Supplementary Material 1 [file 12974_2025_3368_MOESM1_ESM.pdf]

Supplementary Figure 1

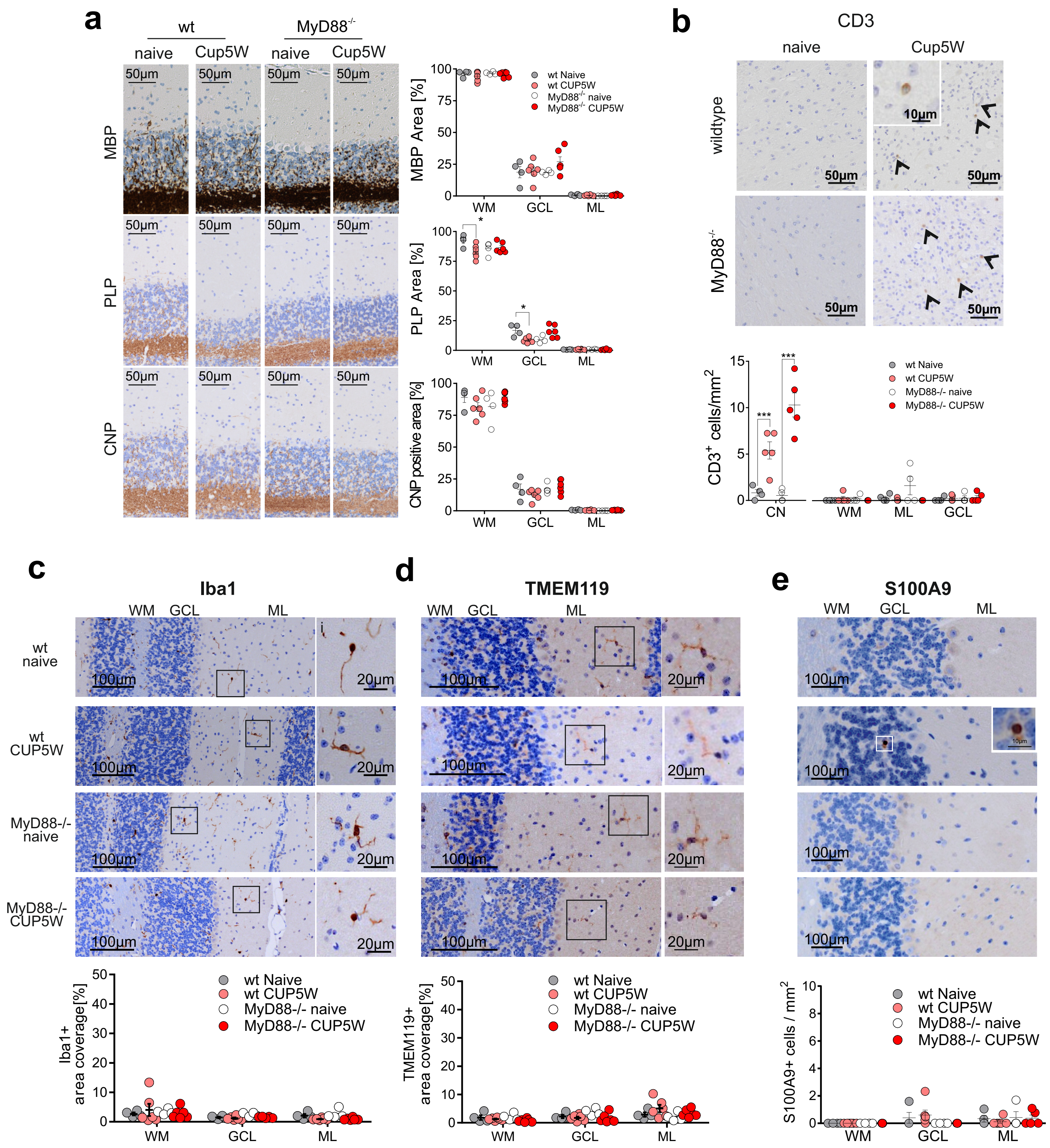

Supplementary Figure 2

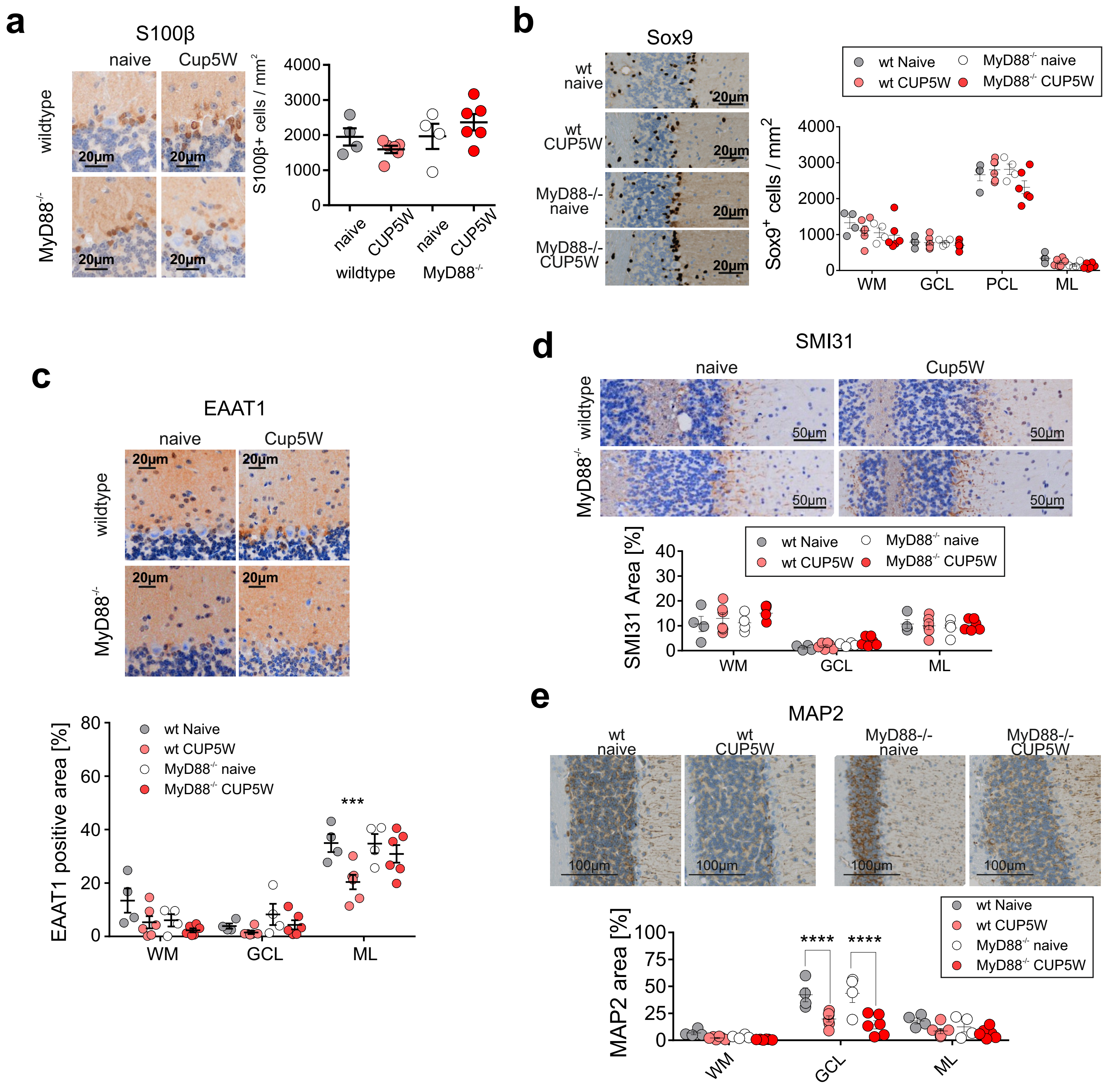

Supplementary Figure 3

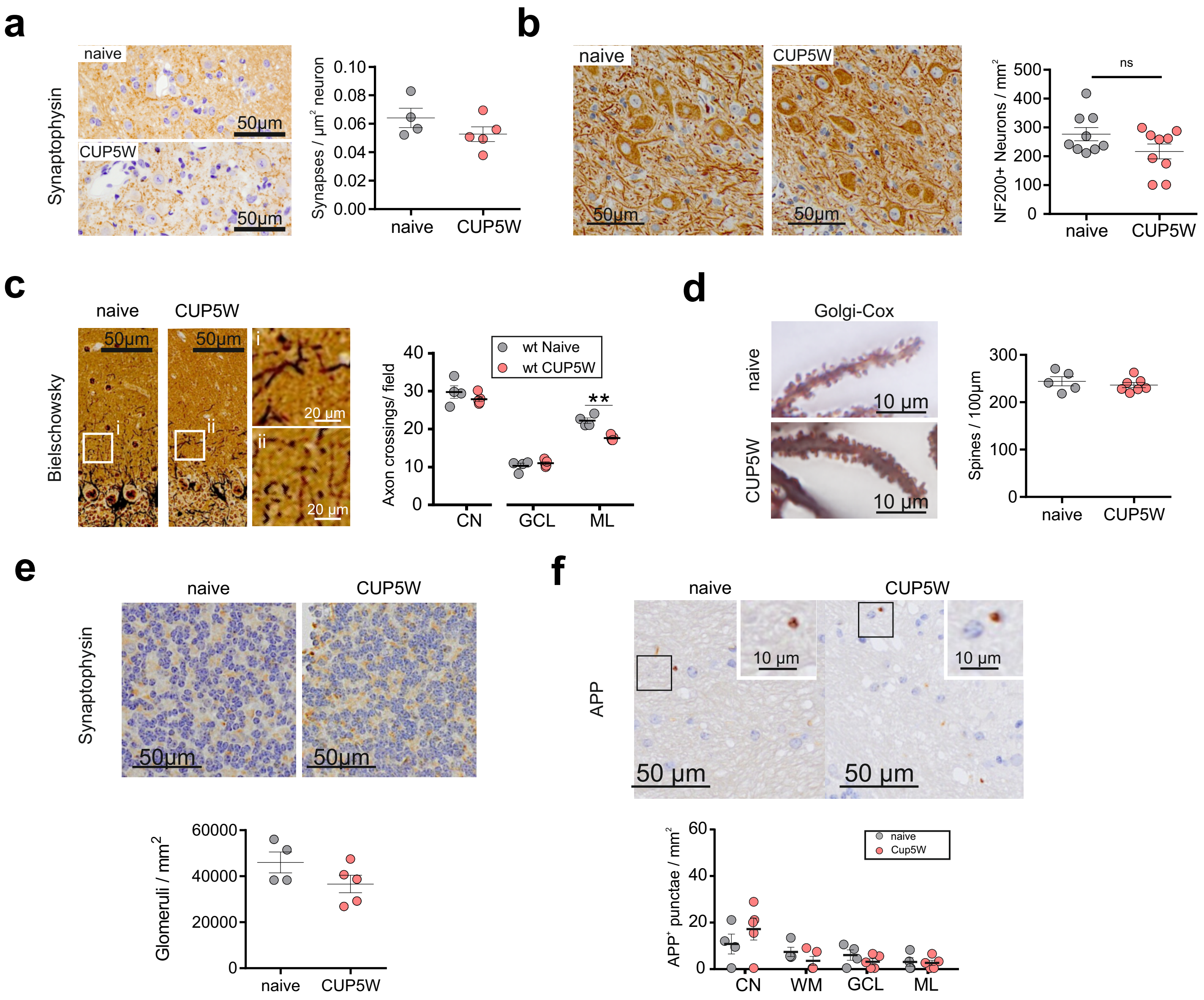

Supplementary Figure 4

a

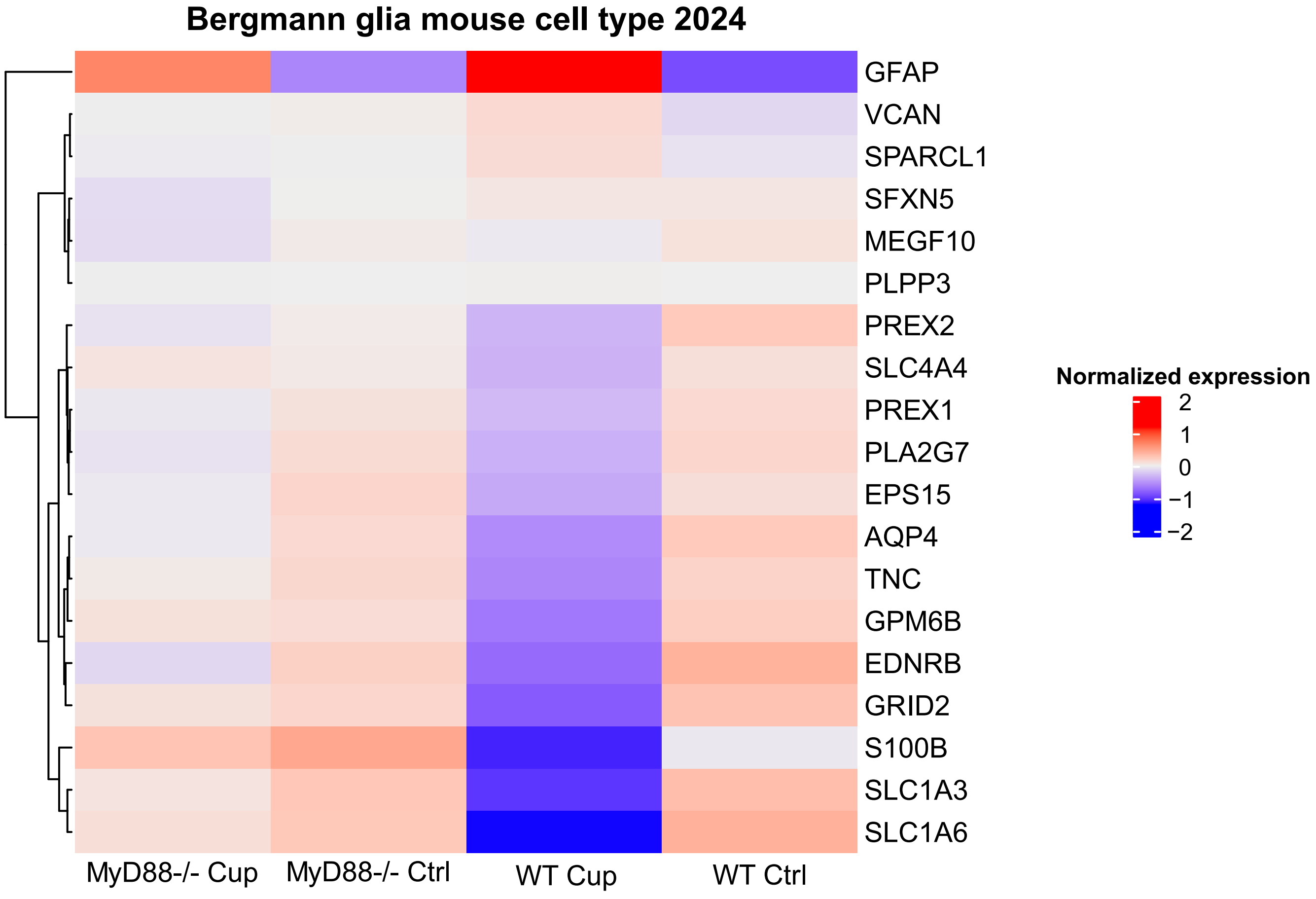

b

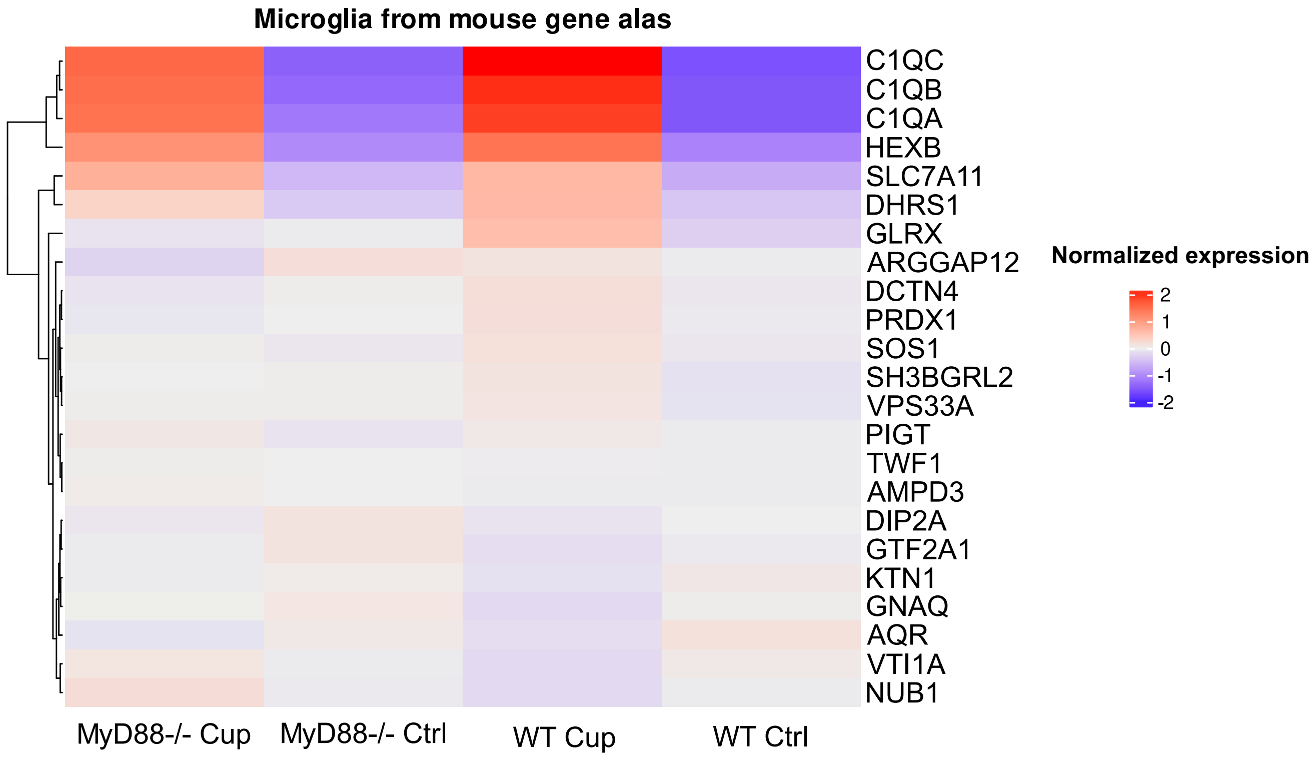

Supplementary Figure 5

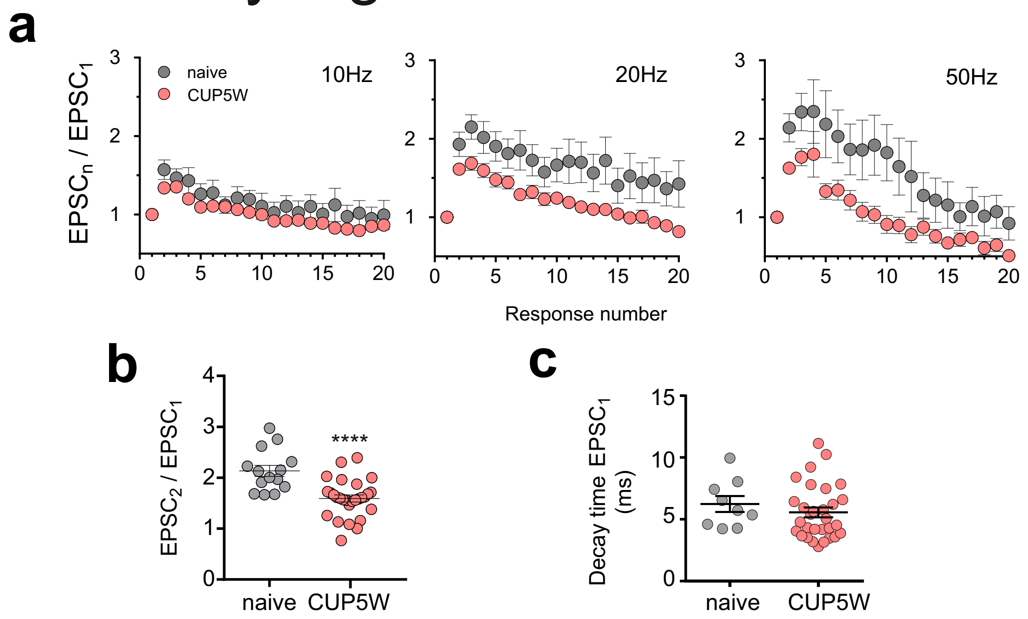

Supplementary Figure 6

a

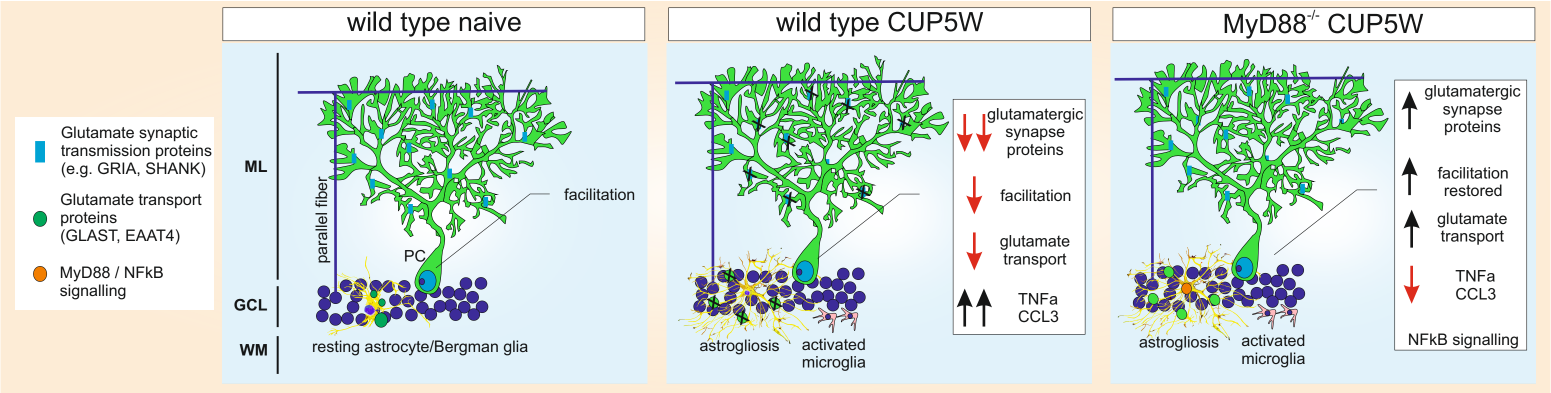

b

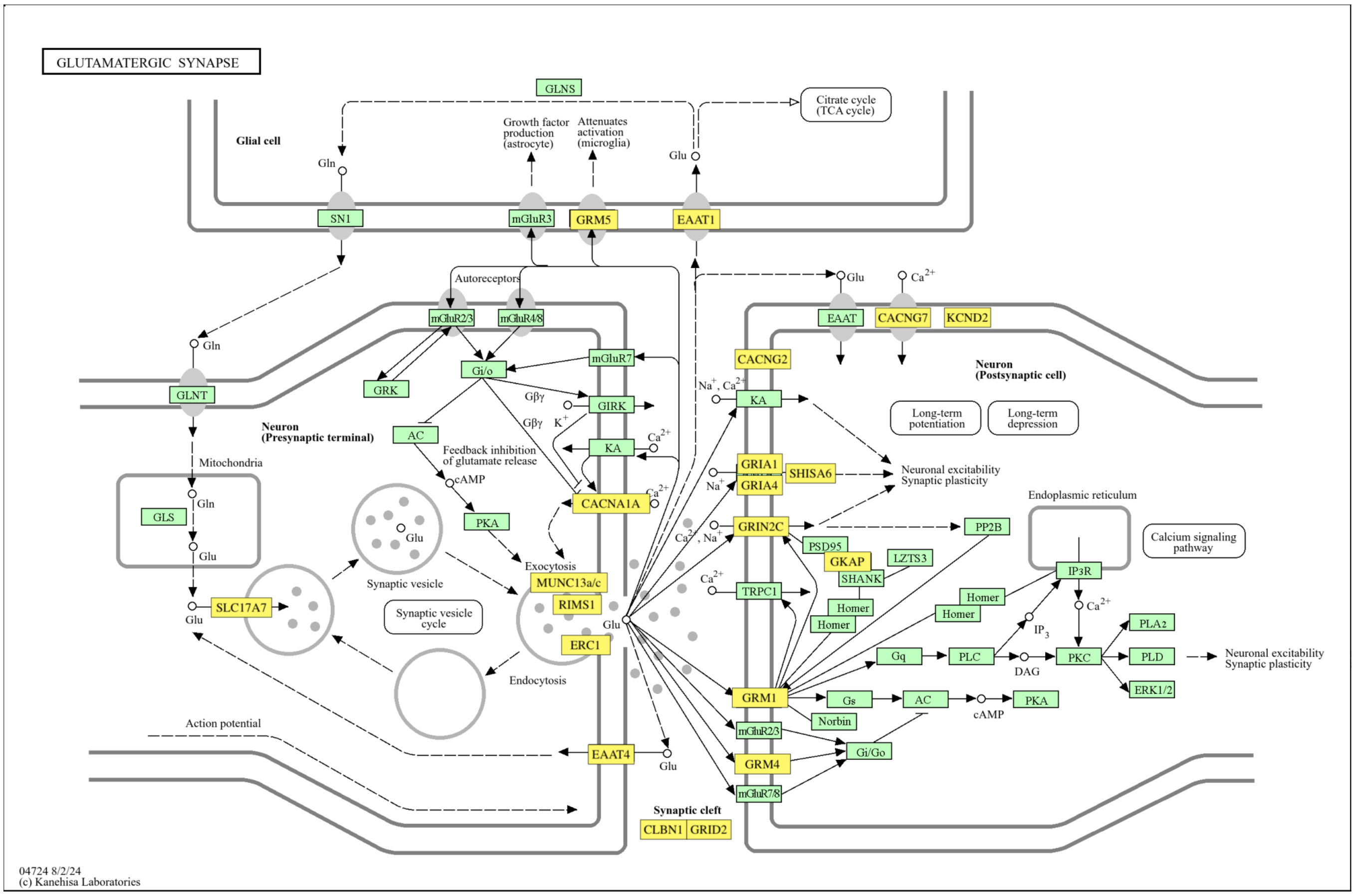

Supplementary Table 1. Gene set enrichment analysis of differentially regulated gene sets in cuprizone-fed as compared to naive wildtype animals

Gene_set	Adjusted P-value	Odds Ratio	Combined Score
mRNA processing (GO:0006397)	2,31E-74	16,55	2938,48
RNA splicing, via transesterification reactions with bulged adenosine as nucleophile (GO:0000377)	6,50E-68	17,69	2866,72
mRNA splicing, via spliceosome (GO:0000398)	1,97E-67	16,20	2600,00
RNA processing (GO:0006396)	5,73E-37	13,45	1212,22
RNA splicing (GO:0008380)	2,63E-29	18,76	1355,03
mRNA metabolic process (GO:0016071)	1,31E-22	15,09	854,38
mRNA 3'-end processing (GO:0031124)	1,08E-19	15,74	783,15
spliceosomal complex assembly (GO:0000245)	1,31E-19	27,86	1377,30
RNA 3'-end processing (GO:0031123)	2,44E-19	17,51	852,94
mRNA transport (GO:0051028)	4,41E-19	11,98	575,14
mRNA export from nucleus (GO:0006406)	1,10E-18	11,49	540,15
RNA export from nucleus (GO:0006405)	8,33E-18	11,13	499,56
mRNA-containing ribonucleoprotein complex export from nucleus (GO:0071427)	8,93E-18	11,72	524,36
regulation of mRNA splicing, via spliceosome (GO:0048024)	6,69E-16	11,57	466,74
RNA transport (GO:0050658)	6,85E-16	13,29	535,08
nuclear export (GO:0051168)	1,23E-13	10,81	378,41
RNA metabolic process (GO:0016070)	3,29E-11	6,82	200,01
gene expression (GO:0010467)	3,40E-11	3,95	115,46
ribonucleoprotein complex assembly (GO:0022618)	5,30E-11	6,63	190,54
regulation of alternative mRNA splicing, via spliceosome (GO:0000381)	1,64E-09	11,61	293,55
mRNA splice site selection (GO:0006376)	3,93E-09	19,25	468,75
RNA splicing, via transesterification reactions (GO:0000375)	4,93E-09	23,21	558,85
chemical synaptic transmission (GO:0007268)	9,13E-09	3,76	87,98
spliceosomal snRNP assembly (GO:0000387)	1,17E-07	13,08	272,51
regulation of cardiac conduction (GO:1903779)	2,04E-07	9,45	191,10
mRNA cis splicing, via spliceosome (GO:0045292)	2,04E-07	32,21	650,38
nucleocytoplasmic transport (GO:0006913)	2,53E-06	10,77	189,88
regulation of neurotransmitter receptor activity (GO:0099601)	3,59E-06	8,01	138,12
termination of RNA polymerase II transcription (GO:0006369)	3,78E-06	12,00	205,97
nucleic acid metabolic process (GO:0090304)	7,42E-06	6,75	110,99
anterograde trans-synaptic signaling (GO:0098916)	8,93E-06	3,43	55,70
regulation of heart contraction (GO:0008016)	8,93E-06	5,64	91,33
glutamate receptor signaling pathway (GO:0007215)	8,93E-06	10,62	171,69
alternative mRNA splicing, via spliceosome (GO:0000380)	1,40E-05	20,01	314,03
import into nucleus (GO:0051170)	1,98E-05	6,09	93,31
cytosolic calcium ion transport (GO:0060401)	5,83E-05	11,86	168,50
regulation of mRNA processing (GO:0050684)	6,22E-05	9,64	136,06
regulation of gene silencing by RNA (GO:0060966)	8,04E-05	7,01	96,73
regulation of posttranscriptional gene silencing (GO:0060147)	8,04E-05	7,01	96,73
nuclear transport (GO:0051169)	8,42E-05	19,43	266,91
regulation of RNA splicing (GO:0043484)	8,69E-05	5,68	77,67
ncRNA export from nucleus (GO:0097064)	9,50E-05	8,95	121,40
regulation of gene silencing by miRNA (GO:0060964)	0,000107821	6,05	81,14
DNA-templated transcription, termination (GO:0006353)	0,00017708	5,73	73,88
regulation of cellular response to stress (GO:0080135)	0,000211332	4,24	53,84
ATP-dependent chromatin remodeling (GO:0043044)	0,000234623	7,83	98,42
positive regulation of synaptic transmission (GO:0050806)	0,000270679	5,44	67,51
spliceosomal tri-snRNP complex assembly (GO:0000244)	0,000294016	21,39	263,15
modulation of chemical synaptic transmission (GO:0050804)	0,000294057	4,33	53,18
calcium ion transmembrane transport (GO:0070588)	0,000378055	4,82	57,88
negative regulation of mRNA splicing, via spliceosome (GO:0048025)	0,000468355	18,72	220,40
regulation of cation channel activity (GO:2001257)	0,000477504	4,69	55,05
regulation of cellular response to heat (GO:1900034)	0,000592759	4,95	56,88
negative regulation of mRNA processing (GO:0050686)	0,000713027	16,64	187,95
positive regulation of RNA splicing (GO:0033120)	0,000997214	8,70	95,17
positive regulation of mRNA processing (GO:0050685)	0,001015893	10,93	119,19

cellular calcium ion homeostasis (GO:0006874)	0,001149759	3,60	38,71
transcription by RNA polymerase II (GO:0006366)	0,001170261	2,53	27,10
neurotransmitter transport (GO:0006836)	0,001174829	4,93	52,85
regulation of NMDA receptor activity (GO:2000310)	0,00117761	8,33	89,09
mRNA 3'-splice site recognition (GO:0000389)	0,001613103	49,80	515,88
endomembrane system organization (GO:0010256)	0,001643789	2,98	30,73
tRNA export from nucleus (GO:0006409)	0,001744252	7,69	78,56
tRNA-containing ribonucleoprotein complex export from nucleus (GO:0071431)	0,001744252	7,69	78,56
regulation of RNA metabolic process (GO:0051252)	0,001744252	5,10	52,10
chromatin organization (GO:0006325)	0,001744252	3,42	34,92
regulation of mRNA polyadenylation (GO:1900363)	0,001928725	12,48	125,84
chromatin remodeling (GO:0006338)	0,001971018	3,95	39,65
protein sumoylation (GO:0016925)	0,001971018	5,56	55,84
cellular metal ion homeostasis (GO:0006875)	0,002160757	3,91	38,79
calcium ion-regulated exocytosis of neurotransmitter (GO:0048791)	0,002361506	17,80	174,70
regulation of RNA export from nucleus (GO:0046831)	0,002361506	17,80	174,70
negative regulation of RNA splicing (GO:0033119)	0,00250253	11,52	112,17
tRNA transport (GO:0051031)	0,002951708	6,89	65,93
histone mRNA metabolic process (GO:0008334)	0,003092402	8,32	79,09
regulation of mRNA metabolic process (GO:1903311)	0,003519429	15,58	145,79
synaptic vesicle exocytosis (GO:0016079)	0,005098336	6,25	56,08
RNA secondary structure unwinding (GO:0010501)	0,005530301	24,90	221,12
signal release from synapse (GO:0099643)	0,008455482	5,71	48,23
positive regulation of histone modification (GO:0031058)	0,008833927	6,72	56,38
DNA topological change (GO:0006265)	0,009291366	19,92	165,80
neurotransmitter secretion (GO:0007269)	0,009621704	5,55	45,96
transmembrane receptor protein tyrosine kinase signaling pathway (GO:0007169)	0,00962231	2,10	17,38
ephrin receptor signaling pathway (GO:0048013)	0,011739512	3,88	31,24
regulation of synaptic transmission, glutamatergic (GO:0051966)	0,012763074	5,26	41,86
dosage compensation (GO:0007549)	0,013504252	16,60	130,20
L-glutamate transmembrane transport (GO:0015813)	0,013504252	16,60	130,20
lipid phosphorylation (GO:0046834)	0,013504252	16,60	130,20
nuclear pore complex assembly (GO:0051292)	0,013504252	16,60	130,20
lamellipodium assembly (GO:0030032)	0,013504252	7,48	58,62
positive regulation of histone methylation (GO:0031062)	0,013504252	7,48	58,62
transcription, DNA-templated (GO:0006351)	0,013586192	2,50	19,56
polyol metabolic process (GO:0019751)	0,013586192	5,12	40,00
inositol phosphate metabolic process (GO:0043647)	0,015522093	5,00	38,26
positive regulation of mRNA splicing, via spliceosome (GO:0048026)	0,015522093	9,58	73,08
synaptic transmission, glutamatergic (GO:0035249)	0,015522093	9,58	73,08
L-alpha-amino acid transmembrane transport (GO:1902475)	0,015522093	7,13	54,27
regulation of cardiac muscle cell contraction (GO:0086004)	0,015522093	7,13	54,27
endoplasmic reticulum organization (GO:0007029)	0,015522093	3,97	30,21
