# Supplementary material for: Astroglial modulation of synaptic function in the non-demyelinated cerebellar cortex is dependent on MyD88 signaling in a model of toxic demyelination
Source: J Neuroinflammation. 2025 Feb 23;22:47. doi: 10.1186/s12974-025-03368-9 (PMC11849172; doi:10.1186/s12974-025-03368-9)
Supplement: Supplementary file 2 — Supplementary Material 2 [file 12974_2025_3368_MOESM2_ESM.docx]

**Supplementary Fig. 1. a.** Panel of representative immunohistochemical stainings for the myelin proteins MBP (top), PLP (middle) and CNP (bottom) and their quantification (right column) showing a significant reduction in wildtype CUP5W in the subcortical white matter (WM) and granule cell layer (GCL). **b.** Representative images and quantification of CD3-IHC showing a significant increase in T cells (arrowheads) in the lesion areas of the cerebellar nuclei (CN) in cuprizone-treated mice while no significant differences were observed in the cerebellar cortex in all conditions. Inset: Higher magnification image of the highlighted region. wt naive n = 4; wt CUP5W n = 5, MyD88^−/−^ naive n = 4, MyD88^−−/−^ CUP5W n = 5. **c.** Representative images of the NAGM immunohistochemically stained for microglia (Iba1), TMEM119 (**d**) and S100A9 (**e**) in naive and cuprizone-fed animals. Morphologically, microglia appeared ramified in all conditions (insets, right column). Bottom panels: Quantification showing no differences in area coverage (Iba1, TMEM19) or cell density (S100A9). Each point represents a measurement from an individual animal (wt naive n = 4; wt CUP5W n = 6, MyD88^−/−^ naive n = 4, MyD88^−/−^ CUP5W n = 6 in a, c-e). Whiskers represent mean ± SEM. P values were obtained after two-way ANOVA followed by Tukey's multiple comparisons test (a-e).Asterisks represent significant p-values (*p < 0.05, **p < 0.01, ***p < 0.001).

**Supplementary Fig. 2. a**. S100β-IHC showing nuclear staining of astroglia in the Purkinje cell layer (left panel). Quantification showing no significant differences in S100β^+^ cell density in any of the conditions studied (right panel). **b.** Representative images of Sox9 immunohistochemistry (left) and quantification (right) showing no significant differences between groups. **c.** EAAT1-IHC (top) showing a significant area reduction (bottom panel) in the molecular layer (ML) in wt CUP5W but not in cuprizone-fed MyD88^−/−^ mice. **d.** Representative images and quantification of area coverage for the neurite marker SMI31. **e**. MAP2 immunohistochemical staining (top) and quantification (bottom) showing a significant neurite reduction in the GCL in cuprizone-treated animals irrespective of genotype. The same dataset for MAP-IHC wt naive and CUP5W is also shown in Fig. 2b. Each point represents an individual animal (wt naive n = 4; wt CUP5W n = 6, MyD88^−/−^ naive n = 4, MyD88^−/−^ CUP5W n = 6). Whiskers represent mean ± SEM. P values were obtained after two-way ANOVA (a-e) followed by Tukey's multiple comparisons test. Asterisks represent significant p-values (*p < 0.05, **p < 0.01, ***p < 0.001).

**Supplementary Fig. 3** **a.** Synaptophysin-IHC of the cerebellar nuclei (left) showing a tendency towards a reduction in synaptic density, which failed to reach statistical significance. Each point represents average values for a single animal (naive n = 4; CUP5W n = 5). **b.** Representative images of the cerebellar nuclei of naive (left) and cuprizone-fed (CUP5W, right) mice immunohistochemically stained against NF200. Quantification of NF200 + neuronal density (right) showing no significant differences between naive (n = 9) and CUP5W (n = 9) animals. Each point represents average values for a single animal. **c.** Representative images of the NAGM stained with Bielschowsky’s silver impregnation. Insets (i,ii) show higher magnification images of highlighted regions. Quantification showing no differences in axonal crossings per field in the cerebellar nuclei (CN) but a significant reduction in the molecular layer (right). Each point represents average values for a single animal (naive n = 4; CUP5W n = 5). High axonal density in the subcortical lobar white matter (WM) is not suitable for quantification with this method and was excluded from the analysis. **d.** Golgi-Cox staining showing dendritic spines of Purkinje cells in the molecular layer. Right: Quantification showing no significant differences. Each point represents average density of spines per animal. A total length > 80 µm per individual was quantified (naive n = 5; CUP5W n = 7). **e.** Representative images (top) and quantification (bottom) of synaptophysin-IHC of the granule cell layer showing no significant differences in the density of glomeruli between naive and CUP5W mice. **f.** Quantification of APP^+^ axonal spheroids showing no increase after cuprizone treatment (right). Each point represents average values for a single animal (naive n = 4; CUP5W n = 5). Whiskers represent mean ± SEM. P values were obtained after Mann Whitney test for a, b, d and e or two-way ANOVA with Sidak’s multiple comparisons for c and d. Asterisks represent significant p-values (*p < 0.05, **p < 0.01, ***p < 0.001, **** p < 0.0001). CN: cerebellar nuclei; WM: white matter; GCL: granule cell layer; ML: molecular layer.

**Supplementary Fig. 4. Differential expression of proteins related to Bergmann glia and microglia. a** Heatmap representation of proteome analysis revealing differential expression of proteins related to Bergmann glia in all conditions studied. Note the rescue effect in MyD88^−/−^ CUP5W animals (first column) as compared to WT-CUP5W (third column). **b.** Similar representation of proteome data for microglial genes showing only small differences between conditions and no apparent rescue effect in MyD88^−/−^ CUP5W.

**Supplementary Fig. 5. Frequency dependent reduction in facilitation in cuprizone-treated mice. a** Quantification of relative EPSC amplitude of current trains showing a frequency dependent reduction in facilitation in CUP5W animals. Each point represents mean normalized EPSC value ± SEM. **b** Relative facilitation as measured in EPSC_2_ (50Hz) in animals fed with cuprizone for one (CUP1W) and 5 (CUP5W) weeks. Each point represents an individual cell. **c.** Decay time constant of Purkinje cell EPSCs in naive (gray) and 5-week cuprizone-fed wildtype mice (pink). Each point represents an individual cell. Whiskers represent mean ± SEM. P values were obtained after Mann Whitney test (b,c). Asterisks represent significant p-values (*p < 0.05, **p < 0.01, ***p < 0.001, **** p < 0.0001).

-

**Supplementary Fig. 6** **Proposed mechanism of synaptic pathology in the cerebellar NAGM in toxic demyelination**. **a.** Cuprizone feeding after 5 weeks (middle panel) leads to a prominent local activation of astrocytes/Bergmann glia (yellow) and discrete microglia activation, with increased expression of inflammatory mediators in demyelinated regions in the cerebellar nuclei e.g. TNFα and CCL3. Under these conditions, reactive astrocytes show a reduced expression of glutamate transport proteins such as GLAST and EAAT4 (green dots). Also, changes in glutamatergic synaptic transmission proteins as compared to physiological conditions (left panel) are observed, which is further accompanied by reduced facilitation of the PF-PC synapse. Cuprizone feeding in MyD88-deficient mice still leads to demyelinated lesion development, astrogliosis and discrete microglia activation, but is characterized by activation of NFkB signaling, a distinct composition of inflammatory mediators and is defined by more subtle changes in protein composition of glutamate transporters and synaptic proteins, without reduction in synaptic facilitation. **b.** Schematic overview of a glutamatergic synapse (modified from KEGG pathway mmu04724). Marked in yellow are proteins which are downregulated after cuprizone feeding in WT animals, but not, or significantly less, in MyD88^−/−^ animals. While some presynaptic proteins are identified, most are components of the postsynapse, including AMPA- and NMDA-receptors, structural proteins and voltage-gated ion channels. Further, the glutamate transports EAAT1 and 4 are among the most differentially expressed proteins between WT and MyD88^−/−^ animals after cuprizone administration. PC: Purkinje cell, ML: Molecular layer, GCL: Granule cell layer; WM: White matter.
